# Supplementary material for: The Therapeutic Relevance of Urolithins, Intestinal Metabolites of Ellagitannin-Rich Food: A Systematic Review of In Vivo Studies
Source: Nutrients. 2022 Aug 25;14(17):3494. doi: 10.3390/nu14173494 (PMC9460125; doi:10.3390/nu14173494)
Supplement: Supplementary file 1 [file nutrients-14-03494-s001.zip › nutrients-1811669-supplementary.pdf]

Supplementary Material:

Table S1. *In vivo* studies assessing the effects of urolithin A, B, UAS03, and methylated urolithin.

Bioactive Properties	Treatment Groups	Intervention (Duration/Dosage/Type of intervention)	Animal Model(s)	Outcome	Reference(s)
**Neuroprotective**<br>***Neurodegenerative disease***	**Control**<br><br>Vehicle<br><br>UroA	**14 days**<br>300 mg/kg<br><br>Treatment	Female APP/PS1 transgenic mice	**<u>Morris water maze</u>**<br>• UroA significantly improved the cognitive function in terms of spatial memory, as seen in the significant difference in escape latency between UroA and vehicle-treated group.<br>• UroA did not influence the locomotor activity of mice.<br>**<u>Probe trials</u>**<br>• UroA treated mice showed significantly more time spent in the target quadrant and increased crossovers compared with vehicle-treated mice.<br>**<u>NeuN-positive cells in the hippocampus</u>**<br>• UroA prevented the loss of $NeuN^+$ immunoreactivity in the CA1 region of hippocampus of APP/PS1 mice.<br>**<u>Terminal deoxynucleotidyl transferase dUTP nick end labelling (TUNEL) assay</u>**<br>• UroA significantly reduced cellular apoptosis in the cortex and hippocampal CA1 of APP/PS1 mice.<br>**<u>Bromodeoxyuridine (BrdU) and doublecortin (DCX) staining</u>**<br>• Significantly more BrdU-positive ($BrdU^+$) cells were seen in the UroA treated mice	[1]

				than in the vehicle treated mice.<br>• Significantly more DCX positive (DCX^+) cells in the dentate gyri of UroA treated mice.<br>**<u>Aβ40 and Aβ42 specific immunochemistry</u>**<br>• UroA significantly decreased the mean area containing Aβ plaques in APP/PS1 mice.<br>• UroA significantly reduced the plaque number/mm^2 of Aβ40 and Aβ42 levels positive plaques compared with APP/PS1 mice.<br>**<u>Quantification of Aβ levels in the cortex and hippocampus with ELISA</u>**<br>• UroA treatment significantly reduced the levels of soluble Aβ40 and Aβ42 in the cortex hippocampus compared with APP/PS1 group.<br>**<u>Antibodies staining against Iba1 and GFAP</u>**<br>• Significantly less intense of reactive astrogliosis and microgliosis in the UroA treated APP/PS1 mice.<br>• Reactive microglia and astrocytes were reduced around Aβ plaques in the brains of UroA treated APP/PS1 mice.<br>**<u>Expression of IL-1β, IL-6, and TNF-*α*</u>**<br>• UroA significantly reduced the levels of the inflammatory mediators IL-1β and TNF-*α* in both the cortex and hippocampus of APP/PS1 mice.	
**Neuroprotective**<br>*Neurodegenerative disease*	**WT (VEH)**<br><br>AD (VEH)	**2 months (APP/PS1 mice)**<br>**1 month (3xTgAD mice)**<br>200 mg/kg/day	APP/PS1 mice<br><br>3xTgAD mice	**<u>Electron microscopy analysis</u>**<br>• UroA and AC stimulated mitophagy and promoted the elimination of defective	[2]

	Actinonin (AC) group (30 mg/kg/day)<br><br>AD(UroA) (200 mg/kg/day)	Treatment		mitochondria in the hippocampus of AD mice.<br>• UroA and AC also normalized mitochondrial morphology and size in the hippocampus of AD mice.<br>**<u>Morris water maze test</u>**<br>• UroA and AC greatly improved learning and memory retention in the AD mice with no difference in swimming speed between groups.<br>• The treatments normalized memory retention in the AD mice to that of the WT mice.<br>**<u>Soluble and insoluble $A\beta_{1-42}$ and $A\beta_{1-40}$ levels in hippocampal tissues</u>**<br>• Insoluble levels of $A\beta_{1-42}$ and $A\beta_{1-40}$, and extracellular Aβ plaque burden were diminished on UroA and AC treatment.<br>**<u>Glial fibrillary acidic protein (GFAP) levels</u>**<br>• UroA or AC did not reduce the GFAP signal in the AD mice.<br>**<u>Soluble and insoluble $A\beta_{1-42}$ and $A\beta_{1-40}$ levels in prefrontal cortex (PFC)</u>**<br>• UroA and AC resulted in the reduction of soluble $A\beta_{1-42}$ and $A\beta_{1-40}$, whereas the insoluble forms remain unchanged in the PFC of AD mice.<br>**<u>OCR evaluation in APOE4/E4 iPSC-derived neurons</u>**<br>• UroA treatment significantly increased the maximal OCR of APOE4/E4 neurons, comparable with that of WT(VEH) neurons.

Genome-wide transcriptomic analysis of hippocampal tissue from WT and AD mice

- UroA treatment restores the transcriptomic profile of AD transgenic mice toward that of WT mice.
- Gene set enrichment analysis of AD(VEH) mice revealed major changes in the expression level of genes associated with inflammation and neuronal function.
- Downregulated in AD(VEH) but increased in UroA treatment: *Gabra2*, *Lrrtm4*, *Slitrk1*, and *Lgi2*.

Western blotting

- UroA and AC treatment significantly increased synaptophysin levels by over 3.5-fold.

Proteolytic cleavage of APP

- UroA did not alter the levels of APP cleavage intermediates, including N-terminal fragments (NTFs) and C-terminal fragments (CTFs), in the hippocampus of transgenic AD animals.
- AC treatment reduced CTFs while no significant effect on the APP NTFs was observed.

Microglial activity

- Increased engulfment of Aβ plaques by microglia in response to AC and UA treatment.
- UroA supplementation but not AC resulted in an increased microglial population.
- UA- and AC-treated animals displayed a

decreased number and length of microglial processes highlighting their shift toward a phagocytic state.

Expression level of proteins involved in microglial phagocytosis and synaptic function in the hippocampus

- UroA and AC increased expression of engulfment-associated protein cluster of differentiation 68 (CD68), microglia-enriched transcriptional regulator interferon regulatory factor 7 (IRF7), and the microglia proliferation marker cluster of differentiation 116 (CD116/CSF2RA), without changing the levels of osteopontin, a cell adhesion and migration regulator.

Mitophagy in microglial cells in the hippocampus

- UroA and AC normalized mitophagy in AD microglia and decreased the extent of mitochrondial damage.

Proinflammatory cytokines

- UroA and AC reduced the protein levels of interleukin-6 (IL-6) and tumor necrosis factor-α (TNF- α).
- UroA treatment increased IL-10 levels in hippocampal tissue fourfold.

Contextual and cued fear conditioning test

- UroA treatment affected a trend of increased cued freezing time in the 3xTgAD mice, while there was no difference in context freezing time.

Object recognition test

				• Normalization of performance by UroA in the 3xTgAD mice to that of WT(VEH) mice.<br>**<u>Y-maze test</u>**<br>• Normalization of performance by UroA in the 3xTgAD mice to that of WT(VEH) mice.<br>**<u>Western blotting</u>**<br>• UroA treatment showed strong p-tau inhibition at sites including Thr181, Ser202/Thr205, and Ser262.<br>**<u>Sex differences in the responses to the mitophagy inducers in both APP/PS1 and 3xTgAD mice</u>**<br>• UroA appeared to be more potent toward male than female APP/PS1 mice.<br>• UroA showed similar effects in male and female 3xTgAD mice.	
**Neuroprotective**<br>***Neurodegenerative disease***	**Normal control group (0.8% Tween 80)**<br><br>Ageing model group<br><br>High dose UroA group (150mg/kg)<br><br>Medium dose UroA group (100 mg/kg)<br><br>Low dose UroA group (50 mg/kg)	**8 weeks**<br>150 mg/kg<br>100 mg/kg<br>50 mg/kg<br><br>Treatment	Male institute of cancer research mice	**<u>Body weight</u>**<br>• Significant decrease in body weight was noted in D-gal-treated model group compared with control group.<br>• Treatment with UroA significantly increased the weight of the mice.<br>**<u>Brain index</u>**<br>• Significant decrease in brain index in D-gal-treated model group compared with control group.<br>• Treatment with UroA significantly increased the brain index of mice.<br>**<u>Biochemical parameters</u>**<br>**AChE and MAO**<br>• Significantly lowered in the whole brain of	[3]

UroA supplemented groups when compared with the whole brain of the model group (all $P<0.01$).

SOD, CAT, GSH-Px, and T-AOC activities and MDA levels in the brain

- Significantly reversed in the decreased in CAT, GSH-Px, SOD and T-AOC activities and increase in MDA levels in brain tissues in UroA groups.

TNF-α, IL-1β, and IL-6 Determination

- The treatment of UroA at different concentrations (50mg/kg, 100mg/kg, 150mg/kg) markedly decreased the levels of 3 proinflammatory cytokines, compared to those of the ageing group activated by D-gal.

Histological findings

H&E staining

- Pyramidal cells, karyopyknosis, disorganized nerve fibers with irregular neurons, and an expended gap of neurons were observed in the CA3 region of D-gal-induced ageing mice.
- UroA treatment reversed these pathological changes and the high dose UroA (150 mg/kg) treatment provided a maximum protective effect with minor changes observed in the morphological structure of the treatment group, identical to that of the normal control groups.

Nissl staining assay

- Neurons were significantly damaged or lost in the CA3 region of the hippocampal

tissue in the model group. Same goes to the population of Nissl bodies in model group mice.

- The number of neurons in the hippocampus treated with urolithin A clearly recovered (*P*<0.01).

<u>Regulation of miR-34a-mediated SIRT1/mTOR signaling pathway</u>

miR-34a in the hippocampal tissue

- The expression of miR-34a was significantly upregulated in ageing mice induced by D-gal compared with that in the control group (*P*<0.01).
- miR-34a expression was notably decreased after the administration of UroA for 8 weeks.

p53/p21

- A positive correlation was observed among p53, p21, and miR-34a and the changes were significantly alleviated by UroA treatment in a concentration dependent manner (all *P*<0.01).

SIRT1

- SIRT1 protein expression was markedly decreased in mice with ageing induced by chronic treatment with D-gal.
- UroA significantly reversed the decreased expression of SIRT1 (all *P*<0.01).

D-gal-induced apoptosis and rescued dysfunctional autophagy

- Cleaved caspase-3 expression significantly decreased, and Bcl-2 expression was notably upregulated in the UroA

treatment group compared with those in the model group (all $P<0.01$).

- The downregulation of autophagy-related proteins was significantly rescued by pre-treatment with UroA (all $P<0.01$).
- The observed increased of p62 level in the hippocampal tissues of ageing mice induced by D-gal was significantly attenuated by treatment with UroA.

Astrocyte activation

- Treatment with UroA significantly downregulated (all $P<0.01$) the GFAP expression and decreased the number of GFAP immunoreactive astrocytes ($P<0.01$) in D-gal-induced ageing mice.

mTOR signaling

- UroA supplementation significantly reversed the increased mTOR expression level.

<u>Effect of UroA on normal young mice and natural ageing mice</u>

- UroA significantly decreased the miR-34a expression levels in both 2 months-old and 12 months-old mice ($P<0.01$ and $P<0.01$).
- UroA upregulated SIRT1 and downregulated p53/p21, suppressed apoptosis and rescue dysfunctional autophagy, attenuate astrocyte activation, and inhibit the mTOR signaling pathway in the hippocampal tissue of normal ageing mice.

**Neuroprotective**<br>*Neurodegenerative disease*	**control group**<br><br>D-gal induced ageing model<br><br>D-gal + 150 mg/kg UroB<br><br>D-gal + 100 mg/kg UroB<br><br>D-gal + 50 mg/kg UroB<br><br><br>**2 months old mice control group**<br><br>2 months old mice + 150 mg/kg UroB<br><br>12 months old mice control group<br><br>12 months old mice + 150 mg/kg UroB	**8 weeks**<br>150 mg/kg/d<br>100 mg/kg/d<br>50 mg/kg/d<br><br>**8 weeks**<br>150 mg/kg/d<br><br>Treatment	Male C57BL/6 mice	**Changes in body weight and BI in ageing mice**<br>• UroB intervention attenuated the decreased in body weight of the mice.<br>• The deteriorate symptoms in the ageing model group alternated in the mice of all doses of the UroB treated groups.<br>**Open field test**<br>• UroB treated mice groups displayed noticeably greater tendency to move further and spent more time in the central areas.<br>**Morris Maze test**<br>• Treatment with UroB significantly restored the poor conditions (increased escape latency).<br>**Spontaneous alternation test (Y-Maze)**<br>• UroB administration significantly increased the time spent and distance travelled in the novel arm compared with ageing mice model group.<br>**Impairment of hippocampal LTP in ageing mice**<br>• The slope of long-term potentiation (LTP) increased in UroB treated group in comparison to the control group.<br>**Biochemical criteria of the whole brain**<br>• UroB supplementation was associated with an increase in the levels of AChE and MAO compared to the model group ($P < 0.05$ or $P < 0.01$).<br>• UroB significantly increased SOD ($p < 0.01$), CAT ($p < 0.01$), GSH-Px ($p < 0.01$),	[4]

and T-AOC (*p* <*0.01*).

- UroB supplementation resulted in a significant decline in MDA levels in the brain tissue of D-gal treated mice (*p* <0.01).
- UroB treatment significantly inhibited the activities of inflammatory cytokines IL-6 (*p* <0.01), TNF-*α* (*p* <0.01), and IL-1β (*p* <0.01).

<u>Hippocampal pathology</u>

- UroB administration reversed the pathological changes such as structural damage, disorganized nerve fibres with irregular neurons and apoptosis-like cells found in the hippocampal of aging mice model group.
- High dose UroB group exerted the maximum protective effect, almost identical to the results of normal control group.
- Similar findings were observed in the cortex of the brain.
- Recovery of the amount of Nissl bodies was observed in the CA3 region of the UroB treated group (*p* <0.01).
- The number of NeuN-immunostained neurons were significantly increased compared to the aging mice model group.
- Pre-intervention with UroB alleviated the decrease in neuronal spines.
- Pre-intervention with UroB alleviated the decrease in neurite arborization.
- The levels of postsynaptic marker PSD95 (*p* <0.01) and presynaptic marker synapsin

I (p <0.01) in the hippocampal tissue were markedly restored in the pre-treatment of UroB.

Inhibition of the activation of microglia and astrocytes

- UroB treated group showed decreased in number of the GFAP-immunoreactive astrocytes and IBA1-immunoreactive microglia.

Advanced glycation

- UroB intervention significantly reduced the formation of AGEs (p <0.01), CML (carboxymethyl lysine) (p <0.01), and CEL (carboxyethyl lysine) (p <0.01) in the brains of aging mice.
- UroB prominently reduced the expression of receptor for advanced glycation end products (RAGE) in aging mice.

Enzyme activity of Cu/ZnSOD and CAT in the brain tissue

- The expression levels of Cu, Zn-SOD, and CAT were dramatically higher in UroB treated group than those in aging mice model group.

Hippocampal neuron apoptosis

- UroB treated group showed remarkably fewer TUNEL-positive neurons in the hippocampus of aging mice.
- UroB treated group showed decreased in the expression of Bax and increased in the expression of Bcl-2.

JNK activation and prevention of cytochrome c release

- UroB treatment group displayed notably higher relative protein expression levels of p-JNK and p-p38 MAPK than the aging mice model group (all p <0.01).
- There was a corresponding decrease in the activity and release of Cyt C from mitochondria into the cytosol after UroB treatment (150, 100, 50 mg/kg) in aging mice.

Photphorylation of Akt and p44/42 MAPK in the mouse brain

- UroB treatment dramatically attenuated the decrease in pAkt in aging model mice (all p <0.01).
- UroB treatment dramatically increased the phosphorylation of p44/42 MAPK in aging mice.

Phosphorylation of Bad in the brains of D-gal treated aging mice

- Phosphorylation at sites Ser112 (p <0.01) and Ser136 (p <0.01) was significantly increased in UroB-treated group.

UroB on normal young mice and naturally senile mice

- The mRNA levels of AGEs, CML, CEL, and RAGE were shown to be decreased in both groups treated with UroB.
- UroB treatment showed an increase in the expression of Cu, Zn-SOD, and CAT in the naturally senile mice compared to the young normal mice model.
- UroB treatment markedly abolished accumulation of cytosolic Cyt C in the

				mitochondria.<br>• UroB downregulated JNK/p38, upregulated PI3K/Akt, improved cognitive deficits, ameliorated structural plasticity, suppressed apoptosis, attenuated astrocyte and microglial activation in the hippocampal of normal aging mice.	
**Neuroprotective**<br>***Autoimmune disease***	**Vehicle**<br><br>UroA	**30 days**<br>10, 25, 50 mg/kg/d<br><br>Treatment	C57BL/6 female mice	**<u>Clinical symptoms of EAE</u>**<br>• 25mg/kg optimized dose to inhibit the progress of EAE.<br>• UroA inhibited disease course during prevention phase, and significantly suppressed the development of EAE at the initial stage.<br>**<u>Immunohistological analysis</u>**<br>• Pronounced inflammatory infiltration and demyelination of the white matter in vehicle-treated group.<br>• The proportion of intact myelin in the spinal cord of UroA-treated group was significantly increased.<br>**<u>Effects of UroA treatment on DCs, CNS mononuclear cells (MNCs)</u>**<br>• The proportion of $CD11c^+$ DCs infiltrating into the CNS was significantly lower than that of the vehicle-treated group.<br>• The proportion of co-stimulatory molecules CD80, CD86, CD40, and CD14 expressed on $CD11c^+$ were remarkedly reduced.<br>• Absolute number (infiltrating macrophages, activated microglia,	[5]

				microglia, and M1-type microglia) of infiltrating cells were significantly reduced.<br>**<u>Migration of pathogenic T cells from the periphery to CNS</u>**<br>• UroA-treated group showed remarkable decreased in the percentages of CD45$^+$, CD3$^+$, CD4$^+$, CD8$^+$ cells infiltrating the CNS.<br>Decreased in Th1 and Th17 cells but no significant differences between UroA and vehicle-treated groups.	
**Neuroprotective**<br>***Opportunistic infection***	**DMSO**<br><br>UroA	**39 days**<br>30 µg<br><br>Treatment	BALB/cJInv female mice	**<u>Cyst formation</u>**<br>• UroA treatment mice survived through the experimental duration, whereas 40% of the infected DMSO control mice succumbed to acute infection and died 10 days post-infection.<br>**<u>Cyst load</u>**<br>• Only mild reduction in the cyst count in the UroA-treated mice comparted to the control mice.<br>**<u>Cyst size</u>**<br>• UroA treated mice have significantly smaller cyst diameter than the DMSO mice (P = 0.0017)<br><br>**<u>Innate response of infected mice towards predatory cat ordor</u>**<br>• No difference in the time spent in the empty dissect was observed during the pre-exposure stage.<br>Upon the introduction of the bobcat urine,	[6]

				UroA-injected mice were less willing to leave the bisect containing their odor and spent less time in the cat odor bisect as compared to the DMSO control mice.	
**Hepatoprotective Nephroprotective**	**control**<br><br>Ageing model (D-gal 150 mg/kg/day)<br><br>D-gal 150 mg/kg/day:<br><br>UroA – 150 mg/kg/day<br><br>UroA – 100 mg/kg/day<br><br>UroA – 50 mg/kg/day	**8 weeks**<br>150 mg/kg/day<br>100 mg/kg/day<br>50 mg/kg/day<br><br>Treatment	Male institute of cancer research mice	<u>**General appearance, body weight and organ indexes**</u><br>**General appearance**<br>• Compared with the control group, the mice in the model group were dull, unresponsive, and their hair was lusterless.<br>• UroA intervention alternated these deterioration symptoms.<br>**Body weight and organ indexes**<br>• 8 weeks of D-gal injection, compared with the control group, there was a significant decrease in the body weight and liver/kidney index in the model group induced by D-gal (*P*<0.05 or *P*<0.01).<br>• UroA intervention attenuated these decreases compared to the ageing group mice (*P*<0.05 or *P*<0.01).<br><u>**MDA content and SOD, GSH-pX, CAT and T-AOC activities in liver and kidney**</u><br>• UroA administration resulted in a decline in the MDA level compared with D-gal alone treated mice (all *P* <0.05 or *P* <0.01).<br>• UroA administration (150, 100 and 50 mg/kg/day) significantly improve the activities of SOD, GSH-Px, CAT and T-AOC in the liver and kidney.<br><u>**TNF-α, IL-1β and IL-6 in liver and kidney**</u><br>• The activities of TNF-α, IL-6 and IL-1β in	[7]

the liver and kidney were significantly upregulated (all $P < 0.01$).

- The mRNA levels of TNF-α, IL-6 and IL-1β in the livers and kidneys of ageing mice induced by D-gal were significantly lowered after the supplementation of UroA (al $P < 0.01$).

Liver and kidney functions

- The levels of ALT and AST were significantly restored in the UroA treatment group (150, 100 and 50 mg/kg/day) compared to model group (both $P < 0.01$).
- All three tested doses of UroA significantly inhibited the increase levels in Cr and BUN ($P < 0.05$ or $P < 0.01$).

Histomorphology of liver and kidney

Liver

- Upon UroA treatment showed an obvious increase in the hepatocellular hydropic degeneration and necrosis compared to the mice in the model group.
- UroA (150 mg/kg/day) appeared very similar in morphological structure to that of the control group.
- UroA treatment noticeably ameliorated the spontaneous activity defects ($P < 0.05$ or $P < 0.01$).

Kidney

- UroA treatment reversed pathological changes such as glomerular basement membrane thickening, tubular necrosis, and cell apoptosis.

				• UroA (150 mg/kg/day) showed maximal protective effect, with minor changes in morphological structure that were almost identical to the normal control group.<br>• The degree of tubular dilation as well as that of the Bowman's capsule were improved by UroA ($P < 0.05$ or $P < 0.01$).<br>**<u>Western blot analysis</u>**<br>The protein expression of cleaved caspase-3 and the Bax/Bcl-2 ratio were significantly downregulated after 8 weeks of treatment with UroA in a concentration-dependent manner (both $P < 0.01$).	
**Nephroprotective**	**Vehicle**<br><br>UroA	**3 days**<br>50mg/kg<br><br>Preventive	C57BL/6 male mice	**<u>Injury markers BUN, NGAL (Neutrophil gelatinase-associated lipocalin), Creatinine, and KIM-1 (Kidney injury molecule-1)</u>**<br>• Significant induction of TFEB nuclear localization with IRI plus urolithin A compared to IRI plus vehicle.<br>• Urolithin A reduced all four cytokine (TNFα, IL1β, MIP1α and MIP2 mRNA levels) levels significantly.	[8]
**Nephroprotective**	**Control**<br><br>IR<br><br>IR+ UroA 20 (20mg/kg/day)<br><br>IR+ UroA 50 (50mg/kg/day)<br><br>IR+ UroA 100	**7 days**<br>20 mg/kg<br>50 mg/kg<br>100 mg/kg<br><br>Preventive	C57BL/6 male mice	**<u>H & E stain</u>**<br>• UroA treatment attenuated tubular cell swelling, interstitial haemorrhage and inflammatory cell infiltration (no significant difference between all 3 doses)<br>• UroA 50 and 100 reduced significantly Cr and BUN levels ((p <0.01 for UroA 50; p <0.001 UA 100)<br>• UroA 20 only reduced BUN (p <0.05)<br>**<u>Apoptosis</u>**<br>• 50mg/kg/day reduced TUNEL-positive	[9]

	(100mg/kg/day)			cells.<br>• Cleaved caspase 3 reduced after UroA treatment (optimal with 50mg/kg/day BOTH (p <0.001)).<br>• UroA 100 did not reduce TUNEL positive cells and CCS-3 as well as UroA 50.<br>**<u>Oxidative stress</u>**<br>• UroA treatment (IR vs. 50 mg/kg/d, 100 mg/kg/d UroA treated IR, p <0.05) attenuated ROS levels.<br>• UroA treatment restore antioxidant enzyme SOD levels (IR vs. 50 mg/kg/d UroA treated IR, p <0.01; IR vs. 100 mg/kg/d UroA treated IR, p <0.01).<br>• UroA 50 and 100 reduced MDA (p <0.05).<br>**<u>p62-Keap1-Nrf2 pathway</u>**<br>• UroA treatment significantly increase Nrf2 levels.<br>• UroA reduced Keap1 expression (suppress Nrf2) (50,100mg/kg; p <0.01).<br>• UroA increase HO-1 (downstream Nrf2 target) (p <0.01 in UroA 20. Non-significant in UroA 50 and p <0.001 in UroA 100).<br>• Increase conversion of LC3 from LC3A to LC3B (IR vs. 20, 50, 100 kg/mg/d UroA treated IR, p < 0.01).<br>• UroA reduce p62 expression (IR vs. 20, 50, 100 kg/mg/d UroA treated IR; p < 0.01).	
**Nephroprotective**	**Control**<br><br>Control (CIS)	**7 days**<br>50 mg/kg body weight<br><br>Treatment	Male Sprague Dawley rats	**<u>Body and kidney weights</u>**<br>• The body weight of control rats significantly increased by 17%. The increase in body weight was marginal in	[10]

	EA+CIS<br><br>UroA+CIS		animals that received CIS with and without intervention (5%).<br>• The relative kidney weights for all CIS groups including those receiving intervention were significantly higher than control animals.<br>**Renal function and histologic findings**<br>• A significant reduction was observed in creatinine levels in rats treated with EA or UroA.<br>• Changes such as degeneration of the tubules leading to cell detachment toward the tubular lumen in CIS controls were not observed in the intervention groups.<br>• Animals treated with EA showed moderate protection in morphological tubular damage, while UroA offered significant protection.<br>**Immunologic findings**<br>• Renal expression of TIM-1 was significantly decreased in rats treated with EA and UroA.<br>• Treatment with EA and UroA displayed decreased in NF-kB expression.<br>• Expression of NOS3 was preserved in renal tubules in the intervention groups.<br>• Rats treated with UroA had dramatically reduced Iba1 expression at the tubular region of the kidney, almost comparable to the negative control animals, while EA failed to attenuate.<br>**Inflammatory findings**<br>• UroA treatment decreased the expression

				of proinflammatory cytokines, such as tumor necrosis factor-α, interleukin (IL)-6, interferon gamma, IL-1α, IL-1β, IL-13, IL-17A, and IL-2. EA treatment failed to reduce these levels.<br>• Both UroA and EA treatments showed a marked increase in the levels of anti-inflammatory cytokine IL-10.<br>• The changes in the cytokine levels were localized in kidney tissue with no systemic inflammatory response was observed in plasma samples reflected by the absence of these cytokines.<br>**Apoptosis evaluation**<br>Treatment with UroA and EA considerably decreased the tubular apoptotic cells in the kidney sections.	
**Nephroprotective**	**Vehicle**<br><br>UroA<br><br>Cisplatin<br><br>Cisplatin+UroA	**5 days**<br>UroA: 100 mg/kg<br>Cisplatin: 25 mg/kg<br><br>Treatment	C57BL/6 male mice	**NGAI, BUN, creatinine, and urinary KIM-1**<br>• Pre-treatment with UroA attenuated all four parameters significantly.<br>**Histological analyses of kidney section**<br>• Significant tubular damage by cisplatin was attenuated by pre-treatment with UroA at 100 mg/kg.<br>**Cisplatin-induced pro-inflammatory cytokine/chemokine followed by leukocyte infiltration in the kidney**<br>• UroA pre-treatment attenuated TNFα, IL-23, IL-18, and MIP2 mRNA expression respectively.<br>• UroA significantly reduced CD11b positive cells.<br>• UroA reduced immune cell infiltration	[11]

				significantly.<br>**<u>Cisplatin-induced damage by determining 4-HNE and nitration modification of renal protein using quantitative ELISA</u>**<br>• UroA pre-treatment almost completely reduced the oxidative stress levels to control vehicle groups.<br>• UroA significantly attenuated cisplatin-induced increased of caspase 3 activity and DNA fragmentation.<br>• UroA reversed cisplatin-mediated depletion of reduced glutathione and increase of oxidized glutathione in mice kidney.<br>• UroA pre-treatment reduced the significant increase in NOX2 mRNA level in the cisplatin-induced mice.<br>• UroA restored the cisplatin-mediated loss in glutathione peroxidase activity and total SOD activity.	
**Nephroprotective**	**Control**<br><br>CIS control<br><br>P2Ns-GA UroA	**19 days**<br>50 mg/kg<br><br>Treatment	C57BL/6J mice	**<u>Effect of nanoparticle UroA on mouse survival</u>**<br>• CIS control group began dying by day 8, with 100% dead by day 15.<br>• Treatment with P2Ns-GA UroA was found to have a positive effect on survival, delaying the onset of death by 1 day in those that died and reducing overall mortality by 63%. Mice that survived in P2Ns-GA UroA group appeared healthy at day 19.<br>• P2Ns-GA UroA group displayed progressively less reduction in weight loss	[12]

after CIS injection and had no discernable changes in kidney mass-to-body weight ratio at euthanasia.

- Blood urea nitrogen and serum creatinine levels of euthanized mice (15-19 days) showed that these parameters had returned to normal for P2Ns-GA UroA treated mice, whereas they were still significantly elevated for the CIS control group.

<u>Histological evaluation of CIS nephrotoxicity and treatment efficacy</u>

- Compared with the negative control and P2Ns-GA UA groups, the CIS control group showed greater interstitial expansion along with increased necrosis and atrophy of the tubules.
- ImageJ analyses of H&E-stained images indicated that the cortical and medullary area occupied by the interstitial space was ~37% in the CIS control group but only 11% and 2% for the P2Ns-GA UA and negative control groups, respectively.
- UroA treatment significantly negated the morphological abnormalities such as increased cross-sectional glomerular diameter, tubular dilation, apoptotic bodies, prominent PAS-positive renal casts within tubular lumens, and thickening of glomerular basement membranes.

<u>Expression of miRNAs and stress-inducible genes</u>

- Kidney-specific reductions in both miR-140-5p and miR-192-5p were observed in the CIS control group at euthansia, whereas these changes were largely reversed with P2Ns-GA UroA.
- Renal expression of NRF2 protein and NRF2-inducible genes [metallothionein 1 (*Mt1*), thioredoxin reductase 1 (*Txnrd1*), and sulfiredoxin 1 homolog (*Srxn1*)] was significantly higher in the CIS control group (8- to 12-fold, 7- to 14-fold, and 4- to 5-fold, respectively) compared with the P2Ns-GA UA and negative control groups.
- P53 protein and P53-inducible genes [cyclin-dependent kinase inhibitor 1A (*Cdkn1a*) (P21), activating transcription factor 3 (*Atf3*), and transformation-related protein 53-inducible nuclear protein 1 (*Trp53inp1*) (SIP)], which in the CIS control group had increases of 7- to 49-fold, 3- to 4-fold, and 9- to 12-fold, respectively, compared with the P2Ns-GA UA and negative control groups.
- Both the CIS control and P2Ns-GA UroA groups exhibited similar increase in the expression of β catenin and transcription factor 7, T cell specific (Tcf7). P2Ns-GA UA did not alter the hypoxic state of the kidney, since both CIS control and P2Ns-GA UA groups had decreased expression of genes that are associated with hypoxia.
- No changes of expression of PARG in any

				group.<br>• Elevated PARP1 expression was observed in the CIS control group.<br>• Reductions in both Bcl-2-to-Bax transcript ratio and intracellular NAD^+ were observed in the CIS control group.<br>• CIS caused marked decreases in mitochondrial complexes I, II, III, and IV. CIS control kidney sections showed increased incidences of TUNEL-positive cells, a sign of apoptosis-induced nuclear fragmentation Renal apoptosis and deficiencies in oxidative phosphorylation were less pronounced in the P2Ns-GA UroA group.	
**Nephroprotective**	**SHAM**<br><br>UUO model<br><br>Enalapril Maleate control group (EM, 20 mg/kg/d)<br><br>UUO+low UroB group (20 mg/kg/d)<br><br>UUO+medium UroB group (40 mg/kg/d)<br><br>UUO+high UroB group (80 mg/kg/d)	**3 weeks**<br>20 mg/kg/day<br>40 mg/kg/day<br>80 mg/kg/day<br><br>Treatment	Sprague Dawley rats	**<u>Psychosis status</u>**<br>• Normal group was active, possessed white and shiny hairs. They also have normal drinking water eating in SHAM group.<br>• UUO group were slouched, and the hair gradually turned yellow and lusterless.<br>• EM group and UroB groups also had similar phenomena to the model group, but the states were improved compared with the model group, especially EM and UUO+high UroB group.<br>**<u>Renal index</u>**<br>• The kidney tissue of normal group rats had a normal appearance, a smooth surface and rosy color.<br>• The size of renal tissues in UUO group were significantly increased compared to	[13]

SHAM group (P <0.01), the surface is uneven, with obvious ecchymosis. The kidney surface is covered with thick adipose tissue, and the renal cortex is obviously thin, with large amount of effusion, and the color is dark red, and the ligature is above.

- EM group and UroB groups also had similar phenomena to the model group, but the states were improved compared with the model group, especially EM and UUO+high UroB group (P <0.05). UUO+low UroB and UUO+medium UroB group exhibited a slight reduction in the size of renal tissue (P <0.05).

Proteinuria

- Concentration of proteinuria markedly increased after modelling (P <0.01).
- EM and UroB groups treatment markedly decreased the concentration of urinary protein as compared to those in the SHAM group at each time point (P <0.05). Especially in the EM group and UUO+high UroB group.

Serum Cr and BUN levels

- Significant elevation in the serum Cr and BUN levels were found in the UUO-induced rats compared with the normal control group.
- EM and UroB groups treatment markedly decreased the levels of Cr and BUN levels (P <0.05), especially UUO+high UroB group (P <0.01).

Histopathological examination

- The renal tubular structure was normal, clear, closely arranged, and there was a small amount of inflammatory cell infiltration, the renal cellular architecture was normal, and the glomeruli maintained a better morphology in the SHAM group at each time point (days 7, 14, and 21).
- Significant renal pathological abnormalities were found in the model group, characterized by severe tubulointerstitial lesions, including tubular atrophy, dilatation, and the increase in the thickness of tubular basement membranes, inflammatory cell infiltration and fibrotic hyperplasia were present in UUO rats. The most severe pathological injuries occurred on days 14 and 21. Visible blue collagen staining and prominent collagen fiber hypertrophy in the renal interstitial were observed in the model group through Masson's staining. The area of blue collagen in the model group increased as time goes by.
- UroB treatment group decreased the tubulointerstitial injury and inflammatory response (all P <0.01). The appearance of blue collagen was significantly improved in the UroB-treated rat kidneys.

TNF-α and IL-6

- The concentrations of TNF-α and IL-6 in serum were significantly increased after modelling (P <0.01).

- TNF-α serum levels were significantly reduced in the rats treated with EM, UUO+medium UroB, and UUO+high UroB (P <0.05). They also significantly decreased the concentration of IL-6 (P <0.05).

MCP-1 protein

- A small amount of MCP-1 protein expression in the rat kidney tissue of normal control group.
- Overexpression of MCP-1 protein was observed in renal tubules, glomeruli, and renal interstitial tissue in the model group.
- In the groups of EM and UroB treatments, the expression of MCP-1 protein in renal tubules and glomeruli was decreased.
- Compared with the model group, the expression of MCP-1 protein in the UUO+high, medium, low, and the EM groups were significantly lower (all P <0.01).

Renal expression of alpha-SMA

- A small amount of red fluorescence was observed in the normal control group.
- Strong red fluorescence was observed in the kidney tissue sections of the model group indicating strong deposition of alpha-SMA distributed in the glomerular capillary vasospasm and part of the mesangial area.
- Compared with the model group, the red fluorescence intensity of renal tissue in each administration group was reduced to

different extents.

- The semi-quantitative analysis showed that the expression of alpha-SMA in the renal tissue of the model group significantly increased compared with the normal control group (P <0.01).
- Compared with the model group, the expression of alpha-SMA in the groups of the UUO+high, medium, low, and the EM were significantly decreased (all P <0.01).

Real-time quantitative PCR

- Compared with SHAM group, the expression levels of TGF-β1, NF-KB p65, Col-IV, Ang II, TNF-α, IL-6 and MCP-1 mRNA in the renal tissue on the 21st day of the model group were significantly increased (all P <0.01).
- Treatment with EM, UUO+high, and medium UroB markedly reduced the expression of TGF-β1, NF-KB p65, Col-IV, Ang II, TNF-α, IL-6 and MCP-1 (all P <0.01).

TGF-β1/Smad expressions

- TGF-β1, Smad2 and Smad3 proteins are rarely expressed in the SHAM group.
- UUO model induced the accumulation of TGF-β1, Smad2 and Smad3 proteins (all P < 0.01).
- EM, UUO+medium, and high UroB treatment significantly inhibited the alterations (all P < 0.01).

TLR4/NF-KB signaling pathway

- TLR4, NF-KB p65, p-IKKα and TRAF6

				proteins expression as drastically increased in rats with UUO as compared to the normal control group (all *P* <0.01).<br>• UroB treatments notably reversed the increased expression of TLR4, NF-KB p65, p-IKKα and TRAF6 proteins in renal tissue of UUO-induced rats (all *P* <0.01).	
**Anti-metabolic**	**High Fat diet**<br><br>High Fat diet with UroA	**12 weeks**<br>20µg/day<br><br>Treatment	C57BL/6 male mice	**<u>Phenotype and blood lipid profiles</u>**<br>**Body weight:** no significant differences.<br>**Total Cholesterol:** significantly lowered in HF+UroA group.<br>**HDL:** no significant differences.<br>**LDL:** significantly lowered in in HF+UroA group.<br>**NEFA:** no significant differences.<br>**Triglycerides:** no significant differences.<br>**Adiponectin:** significantly increased in HF+UroA group.<br>**<u>Glucose level</u>**<br>• HF-UroA mice displayed faster disposal of plasma glucose compared with HF mice.<br>**<u>Insulin level</u>**<br>• HF+UroA mice maintained lower insulin levels than HF mice, despite no differences in glucose levels at 30 minutes post glucose injection.<br>**<u>Insulin tolerance test (ITT)</u>**<br>• HF+UroA mice were significantly more insulin sensitive compared with HF-fed mice.<br>**<u>Glucose tolerance test (GTT)</u>**<br>• HF+UroA mice displayed faster disposal	[14]

of plasma glucose compared to HF mice.

<u>Protein expression pattern for insulin signaling upon acute insulin injection (1 U/kg)</u>

- The phosphorylation status of the downstream targets of insulin (i.e., p-serine/threonine kinase 1 [Akt] and p-insulin receptor substrate 1 [IRS-1]) were significantly higher in HF+UroA mice than HF control mice both in the liver and adipose tissue.

<u>Hepatic triglyceride regulation</u>

- UroA treatment did not induce any apparent morphological changes in the liver compared with control mice.

<u>Transcript levels of *Cyp2e1*</u>

- No differences between treatments group.
- UroA (20μg/day) does not cause hepatic toxicity.

<u>Triglyceride levels</u>

- Significant decrease in HF+UroA mice compared with HF mice.
- H&E staining of the liver tissue revealed triglycerides accumulation was significantly lower in HF-UroA mice than HF mice.

<u>Lipogenic gene and protein expression</u>

- Liver tissue from HF+UroA had decreased levels of lipogenic gene and protein expression (fatty acid synthase, stearoyl-CoA desaturase 1) but significantly increased expression of beta-oxidation genes (carnitine palmitoyltransferase 1

[*Cpt1*] and surtuin 1 [*Sirt1*]).

- Livers from HF=UroA mice had augmented expression of reactive oxygen species-quenching related genes, such as superoxide dismutase 1 (*Sod1*) and 2 (*Sod2*).
- Il1b expression and nuclear factor-κB (NFκB) inhibitor alpha (IκBα) degradation was significantly decreased in HF+UroA liver tissue.
- Livers from HF+UroA mice had significantly reduced levels of the endoplasmic reticulum stress markers of phosphorylated mitogen-activated protein kinase 8 (p-JNK), eukaryotic translation initiation factor 2A (p-EIf2α), and mitogen-activated protein kinase 1 (p-ERK).
- UroA increased microtubule associated protein 1 light chain 3 (LC3II) accumulation, indicating elevated autophagy in the livers of UroA-injected mice.
- mtDNA/nDNA ratio was significantly elevated in HF+UroA livers ($P = 0.002$), indicating augmented mitochondrial biogenesis.

Adipocyte hypertrophy and macrophage infiltration in adipose tissue

- A quantitative analysis of adipocyte size and distribution in the epididymal white adipose tissues (eWAT) showed reduced hypertrophy following UroA treatment.

- FASN and ACC levels were reduced, while peroxisome proliferator-activated receptor gamma (PPARγ) expression was higher in UroA-treated mice.
- The smaller adipocyte size induced by UroA injection also correlated with increased adiponectin gene (*Adipoq*) expression and plasma levels.
- UroA increased expression of PPARγ coactivator alpha (Pgc1a; also known as Ppargc1a) and Sirt1 mRNA in UroA-treated eWAT.
- UroA treatment increased the expression of mitochondrial-specific proteins in eWAT, including voltage-dependent anion-selective channel protein (VDAC) (mitochondrial outer membrane) and pyruvate dehydrogenase (matrix) (PDH).
- Increased mtDNA/nDNA ratio in UroA-treated eWAT compared with vehicle controls.
- Both gene and protein levels of Mcp1 and F4/80 were reduced following UroA injection
- expression of CD11c also known as integrin alpha X (ITGAX), a proinflammatory M1 macrophage marker, was also drastically reduced.

M1/M2 polarization in peritoneal macropahges

- Remarkable decrease in proinflammatory M1 Mϕ markers, including Cd11c, tumor necrosis factor (Tnfα), Il6, Il1b, and Mcp1.

				• The resolving M2 phenotype markers chitinase-like 3 (Ch3l3; also known as Chil3) and macrophage galactose N-acetyl-galactosamine specific lectin 2 (Mgl2) were significantly increased in peritoneal Mϕ.	
**Anti-metabolic**	**LF/LS diet**<br><br>HF/HS diet group<br>- HF/HS only<br>- 0.1% EA<br>- 0.1% UroA<br>- 0.1% EA + 0.1% UroA	**8 weeks**<br>0.1% EA<br>0.1% UroA<br>0.1% of EA and UroA.<br><br>Treatment	Male DBA/2J mice	**<u>Intraperitoneal glucose tolerance test (IPGTT)</u>**<br>• Significant lowered fasting blood glucose levels in UroA fed group, and decreased trend in EA and EA+UroA groups compared with HF/HS group.<br>• Blood glucose levels during IPGTT were comparable among mice fed with HF/HS-diet and HF/HS-diets supplemented with EA, UroA, and EA+UroA.<br>**<u>Intraperitoneal insulin tolerance test (IPITT)</u>**<br>• Fasting blood insulin was similar among all groups.<br>• Blood glucose levels were significantly lower at 15 and 120 mins during IPITT in the EA+UroA group compared with HF/HS group.<br>**<u>Serum Lipids and Adiponectin</u>**<br>• EA, UroA, and EA+UroA significantly reduced serum free fatty acids, while only EA+UroA decreased serum triglycerides.<br>• Significant increase of circulating adiponectin in UroA group.<br>**<u>Proinflammatory Cytokines</u>**<br>• CXCL1 was significantly higher in HF/HS compared to LF/LS diet. While no changes in dietary supplementation of EA, UroA,	[15]

and EA+UroA.

- No changes of MCP1, TNFα, IL6 levels in EA, UroA, and EA+UroA supplementation. MCP1, TNFα, and IL6 in liver, skeletal muscle, and epididymal fat were similar in all experimental groups except MCP1 of skeletal muscle had a higher expression level in HF/HS mice compared with LF/LS mice.

Expression of genes related to lipid metabolism in liver, skeletal muscle, and epididymal fat

- Only *Ucp3* gene expression in SM was significantly increased by dietary UroA supplementation compared with HF/HS mice.
- Expression levels of other gene were not altered by EA, UroA, and EA+UroA.
- Expression levels of lipolysis marker *Lipe* and *Pnpl2* were not altered by EA, UroA, EA+UroA.

Markers of Mitochondrial Function

- UroA supplementation significantly increased mitochondrial density in skeletal muscle compared with HF/HS group.
- Uro-A and EA+UroA supplementation significantly increased Mfn2 gene expression in both liver and skeletal muscle.
- Uro-A supplementation significantly increased the gene expression of mitophagy markers Prkn and Pink1 in

				both skeletal muscle and liver.<br>• Other mitochondrial biogenesis markers, Errα and Pgc1α were not changed among experimental groups.<br>• All the mitochondrial markers were similar in all experimental groups for epididymal fat.<br>**<u>Primary hepatocyte mitochondrial respiratory capacity</u>**<br>• EA and EA+UroA significantly decreased proton leak in primary hepatocytes from HF/HS mice.<br>• EA, UroA, and EA+UroA had no effect on basal, maximal and ATP linked OCR of primary hepatocytes from HF/HS mice.	
**Anti-metabolic**	**Control (normal chow diet)**<br><br>Model (high fat diet)<br><br>UroA (high fat diet)	**8 weeks**<br>50 mg/kg<br><br>Treatment	Specific pathogen free (SPF) grade male C57BL/6 mice	**<u>Expression of apoptotic proteins</u>**<br>• The expression of cleaved-caspase 3 and cleaved-caspase 1 was markedly decreased after treatment with UroA (P <0.01).	[16]
**Anti-metabolic**	**Control**<br><br>Diabetic model<br><br>UroA treated<br><br>UroA and chloroquine co-treated group	**14 weeks**<br>50 mg/kg/day<br><br>Treatment	C57BL/6 male mice	**<u>Body weight</u>**<br>• The body weight curve began to rise after UroA treatment for 2 weeks, but not statistically different at the end of the experiment.<br>• The bodyweight of UroA and chloroquine co-treated mice was significantly lower than that of diabetic mice and UroA-treated mice (P <0.05) at the last two weeks (12th and 14th week).<br>• After 8 weeks of treatment	[17]

- Diabetic model group: decreased by 8.2%
- UroA group: decreased by 3.6%
- UroA+CQ group: decreased by 13.3%

Water and food consumption, and urine produced

- Food and water consumption as well as the volume of urine produced by the diabetic mice were higher than that of normal control mice (*P* <0.01).
- Statistically significant improvement was observed in UroA treatment group for 2 weeks until the end of the experiment (*P* <0.05).
- No difference between UroA+CQ mice and the diabetic model group.

Plasma FBG (Fasting Blood Glucose), glucose tolerance, and GHb (Glycated hemoglobin) level

- Plasma FBG level in diabetic mice increased after 6 weeks feeding on high-fat diet (*P* <0.01), which reached 20.6±0.5 mmol/L after STZ injection, reflecting an overall increase of 10.2%.
- UroA treatment for 1 week significantly decreased FBG and GHb level (*P* <0.01), FBG level decreased by 21.7%.
- FBG and GHb level in UroA+CQ co-treated mice remained high throughout the experiment and the level of FBG and GHb in this group was not different from that of diabetic mice.

- Diabetic mice showed poor glucose tolerance, having significantly higher glucose level at 0-120 min time points ($P < 0.01$).
- UroA remarkably decreased glucose level at the 60 min and 120 min time points after glucose loading.
- UroA+CQ mice showed poor glucose tolerance, with significantly higher glucose level at 120 min time point.

<u>Plasma C-peptide level</u>

- Significant increased ($P < 0.01$) in plasma C-peptide level, while significant decreased of HOMA-β index were observed in diabetic mice ($P < 0.01$).
- UroA treatment decreased C-peptide level ($P < 0.05$) and increased HOMA-β index value markedly ($P < 0.01$).
- UroA+CQ mice showed no difference compared to UroA-treated mice. But HOMA-β index value was significantly decreased in UroA+CQ mice.

<u>Plasma MDA and GSH level</u>

- Plasma MDA level significantly higher in while plasma GSH level was lower in diabetic mice compared to normal control mice ($P < 0.01$).
- UroA treatment decreased MDA and increased GSH level significantly ($P < 0.01$).
- UroA+CQ showed increased in plasma MDA, while a reducing trend in the plasma GSH level compared to UroA group.

Plasma IL-1β, IL-10, and TNF-α levels

- Plasma IL-1β and TNF-α level were increased while IL-10 level was significantly decreased in diabetic mice ($P < 0.01$, $P < 0.05$).
- UroA treatment decreased IL-1β, TNF- α level and increased IL-10 level significantly ($P < 0.05$).
- No statistical difference in plasma IL-1β, IL-10, and TNF-α levels in UroA+CQ mice compared to UroA group.

Histopathology

- Islets of diabetic model animals showed severe structural disruption, as well as reduced islets' size and relatively decreased number of islets.
- The severity of the structural disruption, as well as reduce islet's size and relatively decreased number of islets were reduced in mice treated with UroA.
- Histopathological changes of pancreatic islets of diabetic mice co-treated with chloroquine and UroA were similar with those of the diabetic model group.

Transmission Electron Microscopy (TEM)

- Fewer β-cells were observed in the islet of diabetic mice, less secretory granules, and more swollen mitochondria, as well as endoplasmic reticulum expansion and formation of myelin sheath body.
- The level of pancreatic ultrastructural changes was lower in UroA-treated mice compared with diabetic mice model mice.

Many empty autophagic vacuoles with fully degraded organelle fragments were formed in β-cells of UroA-treated mice.

- In UroA+CQ treated mice, the mitochrondria changes in pancreatic cells such as swelling, unclear double-layer membrane structure, and internal crest fracture were almost similar to those of diabetic model group. The number of autophagic vacuoles with partially degraded organelle fragments detected in β-cells of mice that received UroA+CQ treatment was higher.

Microtubule-associated protein conversion, p62, beclin1, and ATG5 expression

- Conversion of microtubule-associated protein 1 light chain 3-I (LC3-I) to LC3-II and p62 expression were increased while beclin1 and ATG5 expression were decreased in pancreas of diabetic model mice ($P < 0.01$).
- UroA treatment further increased LC3-II/I ratio ($P < 0.05$), decreased p62 protein expression, increased belin1 and ATG5 expression ($P < 0.05$, $P < 0.01$) compared with diabetic model mice.
- UroA+CQ restored the LC3-II/I ratio, p62 and beclin1 expression in pancreas to the level of diabetic mice, while the expression level of ATG5 had no statistically difference compared to UroA-treated mice.

Cleaved caspase 3 expression

				• Compared to normal mice, cleaved caspase 3 expression increased greatly in the pancreas of diabetic model mice (P <0.01).<br>• UroA treatment decreased cleaved-caspase 3 expression (P <0.05).<br>• UroA+CQ treatment decreased the cleaved-caspase 3 expression.<br>**<u>AKT/mTOR signal pathway</u>**<br>• Diabetic mice showed decreased phosphorylated AKT level (p-AKT ser473) and phosphorylated mTORC1 (p-mTOR ser2448) levels in pancreas compared with normal control mice (P <0.01).<br>• UroA treatment increased p-AKT and p-mTORC1 levels compared with diabetic model mice.<br>• UroA+CQ co-treated mice showed decreased p-AKT and p-mTORC1 levels compared to UroA group.<br>There was no significant difference in pI3Kp85, AKT and mTORC1 levels among the groups.	
**Anti-metabolic**	**Normal diet (ND)**<br><br>High-fat diet (HFD)<br><br>HFD+UroA<br><br>HFD+UroB	**4 weeks**<br>2.5 mg/kg<br><br>Treatment	Wistar male rats	**<u>Body weight</u>**<br>• Final body weight of HFD group was significantly higher than the weight of NFD animal (P <0.05).<br>• No significant difference in weight between ND group and HFD with UroA or UroB group.<br>• HFD+UroA (19%) and HFD+UroB (21%) showed a significant (P <0.05) reduction in the final body weight when compared	[18]

				with untreated animals fed on an HFD.<br>**<u>Serum lipid levels</u>**<br>• Compared with ND, HFA resulted in a significant increase in the serum cholesterol (*P* <0.01), triglycerides (*P* <0.001) and LDL-C (*P* <0.05) levels and a significant reduction in the HDL-C (*P* <0.05) level.<br>• HFD+UroA group showed a significant decrease in serum cholesterol (*P* <0.01), triglycerides (*P* <0.001) and LDL-C (*P* <0.05) levels and a significant increase in the HDL-C (*P* <0.05) level when compared with HFD group.<br>• HFD+UroB showed a significant reduction in the levels of serum cholesterol (*P* <0.01), triglycerides (*P* <0.001), LDL-C (*P* <0.01) and a significant increase in the level of HDL-C (*P* <0.05).<br>• No significant difference in all the measured serum biochemical parameters between NFD group and HFD+UroA or HFD+UroB.<br>• No significant difference was observed in the serum biochemical parameters between HFD+UroA and HFD+UroB treated animals.	
**Anti-metabolic**	**Normal control group**<br><br>High fat model group	**8 weeks**<br>50 mg/kg<br><br>Treatment	C57BL/6 male mice	**<u>High-fat diet induced pancreatic inflammation</u>**<br>• UroA intervention supressed TXNIP protein levels compared to model group (*p* <0.05).<br>• UroA intervention significantly reduced	[19]

	High fat + UroA group			the levels of NLRP3 inflammasome (*p* <0.01).<br>• UroA intervention significantly suppressed the level of IL-1β (*p* <0.01) and TNF-*α* (*p* <0.01), and increased IL-10 (*p* <0.01) in pancreas of the high fat diet mice group.<br>**High-fat diet induced ER stress and energy stress in the pancreas of diabetic mice**<br>• UroA treatment significantly downregulated phosphor-PERK in pancreatic tissues compared to the model group (*p* <0.01).<br>• UroA treatment further upregulated the levels of AMPK in model group (*p* <0.01).	
**Anti-metabolic**	**Control (50% ethanol)**<br><br>UroB	**14 days**<br>10 mg/kg/day<br><br>Treatment	apoE$^{-/-}$ mice	**Liquid plaque deposition in atherosclerotic mice**<br>• UroB treatment reduced the lipid plaque deposition in mice by half compared to the control group. Obvious lipid deposition was noted in the plaques of the control group compared to UroB treated group in the staining with Oil Red O.	[20]
**Anti-tumour**	**Vehicle (Sunflower oil)**<br><br>UroA	**4-5 weeks**<br>50mg/kg<br><br>Treatment	BALB/c athymic male mice (nu/nu)	**Xenograft studies**<br>• Inhibition of tumour growth: Treatment of UroA inhibited tumour growth in both PC-3 and C4-2B xenografts. Inhibition of C4-2B tumours was significant and the tumours were more sensitive to UroA compared to tumours raised from PC-3 cells.<br>**Expression of tumour markers:**<br>• C4-2B tumour: The reduction in the	[21]

				expression of AR, Ki-67, and pAKT was significant (P = 0.0004), (P = 0.01), (P = 0.0008), in UroA treated C4-2B tumours (P = 0.04) compared with the vehicle.<br>• PC-3 tumour: The reduction in Ki67 was significant (P = 0.04) while reduction observed in pAKT was not significant (P > 0.05) in the UroA treatment compared with the vehicle treatment.	
**Anti-tumour**	**Vehicle**<br><br>UroB (40mg/kg)	**30 days**<br>40 mg/kg<br><br>Treatment	mice	**<u>Suppression of tumor growth in vivo with subcutaneous xenograft assay</u>**<br>• UroB treatment with intraperitoneal injection or subcutaneous injection strikingly decreased the average tumor volume and average tumor weight.<br>• At 30 days after the inoculation, the control group carried larger burdens compared with treated groups.<br>Further immunohistochemistry (IHC) analyses revealed that Ki-57, a classical marker of cell proliferation was significantly reduced in UroB treated groups.	[22]
**Anti-tumour**	**Control**<br><br>mUA groups<br>20 mg/kg group<br><br>40 mg/kg group<br><br>80 mg/kg group	**4 weeks**<br>0 mg/kg<br>20 mg/kg<br>40 mg/kg<br>80 mg/kg<br><br>Treatment	BALB/c mice	**<u>DU 145 xenografts in Balb C nude mice</u>**<br>**Mean tumor volume**<br>• Control: 800 mm^3<br>• 20 mg/kg: 600 mm^3<br>• 40 mg/kg: 480 mm^3<br>• 80 mg/kg: 410 mm^3<br>**Micro-RNAs**<br>• miR-21 was suppressed by mUA treatment<br>**Western blotting**<br>• Protein expression PTEN was elevated by	[23]

				mUA (80 mg/kg).	
**Myocardial protective**	**Control (Sham)**<br><br>I/R (0.5 ml dimethylsulfoxide)<br><br>I/R+UroA	**1 day prior to disease induction.**<br>1 mg/kg<br><br>Preventive	C57BL/6 male mice	**Myocardial infarct size**<br>• Infarct size in the I/R+UroA group was markedly reduced compared with I/R group.<br>**TUNEL assays**<br>• Pre-treatment with UroA significantly prevented cardiomyocyte apoptosis in mice subjected to I/R.<br>**Echocardiographic examinations**<br>• LVFS and EF were significantly lower in I/R group compared with Sham group, which were improved by UroA pre-treatment.<br>**Serum CK and LDH activities**<br>UroA pre-treatment effectively blocked the increase of CK and LDH activities compared with I/R group.	[24]
**Myocardial protective**	FUNDC1$^{f/f}$<br>- **PBS (sham-operated control)**<br>- LPS<br>- UroA<br><br>FUNDC1^{CKO}<br>- **PBS**<br>- LPS<br>- UroA	**48 hours**<br>30 mg/kg<br><br>Treatment	FUNDC1$^{f/f}$ mice<br><br>cardiomyocyte-specific FUNDC1 knockout (FUNDC1^{CKO}) mice	**FUNDC1$^{f/f}$ mice**<br>• Administration of UroA suppressed myocardial injury biomarker levels (LDH, Troponin T, and CK-MB.).<br>• Normalized cardiac function, including LVEF, LVDd, and FS were observed in mice pretreated with UroA.<br>**FUNDC1^{CKO} mice**<br>• UA failed to suppress cardiac injury biomarker levels in FUNDC1^{CKO} mice.<br>• LPS-induced cardiac dysfunction could not be markedly reversed by UroA in FUNDC1^{CKO} mice.<br>**UPR^{mt}**<br>• UroA treatment appeared to partly	[25]

				prevent UPR^{mt} activation in LPS-treated mice.<br>Activation of UPR^{mt} partly reduces sepsis-induced myocardial injury and mitochrondrial dysfunction in FUNDC1-knockout mice.	
**Myocardial protective**	**Control (non-diabetic)**<br><br>Control + urolithin A (2.5mg/kg/ip)<br><br>STZ<br><br>STZ + Urolithin A<br><br>STZ+Urolithin A + Ex-527 (1mg/kg/SIRIT1 inhibitor)	**8 weeks**<br>2.5mg/kg<br><br>Treatment	Kyoto Wistar male rats	ALL (*p* <0.05)<br>**<u>Metabolic markers, hemodynamic, cardiac markers</u>**<br>• Compared to STZ-treated rats, UroA treated rats:<br>o Heart weight, heart index, serum levels CK-MB and Troponin –1, LVEDP significantly reduced.<br>o Levels of LVSP, dp/dtmax and dp/dtmin significantly increased)<br>o No effect on glucose and insulin.<br>**<u>LV, Collagen deposition and Fibrotic markers</u>**<br>• UroA compare with STZ treated group:<br>o Normal LV architectures, less collagen deposition, lower mRNA levels of TGF-b1, Smad2, and Col1A1<br>**<u>Markers in oxidative stress and antioxidants</u>**<br>• UroA in control or diabetic rats (STZ treated)<br>o reduced ROS, MDA; increased GSH, SOD and mRNA levels of Nrd2, H0-1 and nuclear protein levels of Nrf2 in LV compared with control/STZ treated<br>**<u>Inflammatory markers</u>**<br>• UroA treatment in control and STZ treated	[26]

				rats<br>o no significant changes in cardiac mRNA levels of NFκb but significant reduction in TNF-α, IL6, nuclear protein level NFκb p65 compared with control and STZ group<br>**<u>Intrinsic cell apoptosis</u>**<br>• UroA treated diabetic mice compared with STZ mice:<br>o Reduce mRNA levels of Bax, Cleaved caspasepe3, protein levels of cytochrome C sig reduced while, enhanced protein levels of Bcl2 in left ventricular (LV) of UroA treated diabetic mice.<br>**<u>SIRTI</u>**<br>• Significant increase in SIRT1 (mRNA, nuclear activity, total/ nuclear protein levels) in LV of both control UroA and STZ UroA compared with control and STZ.<br>• All results were reversed in EX-527 Tx.<br>**<u>Transcription factor acetylation</u>**<br>• UroA (control UroA and the STZ UroA) groups lowered the levels of acetyl-FOXO1, Nrf2, NF-kB, and p53 compared with groups without UroA treatment. All these benefits of UroA were prevented by Ex-527.	
**Myocardial protective**	**Sham**	**2 weeks**<br>MI+UroB (2.5 mg/kg/day)	Sprague Dawley male rats	**<u>Echocardiography, haemodynamics and BNP test</u>**	[27]

	MI<br><br>MI+UroB (2.5 mg/kg/day)<br><br>MI+UroB (5 mg/kg/day)	MI+UroB (5 mg/kg/day)<br><br>Treatment	• UroB inhibited the increase in HR and the prolongation of PR interval post-MI without affecting the P wave duration or QRS width.<br>• 2.5 mg/kg/day UroB had no significant effect on LVEDD, EF%, FS% or dP/dt_{min} after MI in rats. The only benefits were upregulation of dP/dt_{max} and inhibition of the secretion of BNP.<br>• 5 mg/kg/day UroB comprehensively inhibited the deterioration of these cardiac function indexes (LVEDD, EF%, FS%, haemodynamics, and BNP).<br>**Langedorff-perfused heart system**<br>• UroB notably reduced the susceptibility to ventricular tachyarrhythmia after MI (20%).<br>**Masson's trichrome staining**<br>• UroB treated hearts exhibited significantly smaller necrotic areas, such as reduced infarct sizes, compared to the MI group.<br>**H&E and WGA staining**<br>• UroB treated hearts showed a decrease in myocyte size in the myocardium of the infarct border zone (IBZ).<br>**mRNA expression of collagen I, α-SMA, TGF-β1 and CTGF**<br>• UroB treatment reversed the alterations of increased mRNA expression of collagen I, α-SMA, TGF-β1 and CTGF in the IBZ.<br>**Immunofluorescence staining of CD68 marker**<br>• UroB treatment for 2 weeks significantly

				attenuated $CD68^+$ macrophage infiltration through decreased expression of CD68 mRNA.<br>**<u>mRNA and ELISA testing of pro-inflammatory markers</u>**<br>• UroB treatment induced the downregulation of TNF-α and IL-6 at mRNA and protein levels.<br>• UroB increased the gene expression of IL-10, but it did not notably affect the protein expression of IL-10.<br>**<u>JAK/STAT3 and Smad2/3 signalling</u>**<br>• UroB treatment markedly inactivated the expression of phosphorylated JAK/STAT3 and Smad2/3.	
**Myocardial protective**	**Sham group**<br><br>IR surgery group (control)<br><br>IR+UroB group	**2 days**<br>0.7 mg/kg<br><br>Treatment	Sprague-Dawley male rats	**<u>Hemodynamic parameters of ventricular function</u>**<br>• UroB treatment significantly alleviated the impairment during both ischemia and reperfusion period.<br>**<u>Serum CK and LDH</u>**<br>• UroB treatment lowered the release of serum CK and LDH.<br>**<u>H&E staining and Evans blue-TTC staining</u>**<br>• UroB treatment had no significant effect on area at risk, it significantly reduced the infarct size from 41.5% to 9.3%.<br>**<u>TUNEL staining</u>**<br>• The significant increase of TUNEL-positive cells in IR group compared to Sham group was found to be reduced by UroB treatment.<br>**<u>Cleaved caspase 3</u>**	[28]

				• The increased in IR group was found to be reduced by UroB treatment.<br>**<u>DHE assay</u>**<br>• The level of overall superoxide anion radicals was increased significantly in IR group, which was reduced by about 50% (from 18.93 to 9.26 positive cells/0.01 mm^2; *P* <0.001) after UroB treatment.<br>**<u>MDA and SOD level</u>**<br>• UroB significantly reduced lipid peroxidation product MDA level and restoring antioxidant enzyme SOD level.<br>**<u>LC3 and p62 levels</u>**<br>• IR increased the ratio of LC3II/I and decreased p62 level, which was significantly reversed by UroB treatment.<br>**<u>Phosphorylation of upstream mTOR at Ser2448 and ULK1 at Ser757</u>**<br>• UroB treatment downregulated the phosphorylation of upstream mTOR at Ser 2448 and ULK1 at Ser757.	
**Anti-inflammatory**	**Vehicle**<br><br>UroA<br><br>EA<br><br>Indomethacin	**8 hours**<br>40 mg/kg bodyweight<br><br>Treatment	C57BL/6 mice	**<u>PMA (phorbol 12-myristate 13-acetate)-induced ear edema and MPO (Myeloperoxidase) activity</u>**<br>• Mice treated with UroA displayed significantly reduced PMA-induced ear edema by 43% when compared to vehicle-treated mice.<br>• The extent of protection against PMA-induced ear edema mediated by UroA is almost comparable to indomethacin which remarkedly reduced ear edema by 47.5%.<br>• EA failed to provide significant reduction	[29]

				on PMA-induced ear edema.<br>• UroA and EA significantly inhibited the MPO activity induced by PMA, but the inhibition was mediated at a greater extent by UroA than EA. Indomethacin also decreased the MPO activity.<br>• H&E images of ear edema suggest decreased inflammatory cells in UroA-treated animals compared to EA treatment or vehicle. The images also suggest UroA treatment also reduced swelling of ear caused by PMA treatment.	
**Anti-inflammatory**	**Naïve**<br><br>UroA<br><br>Placebo	**6 days**<br>0.114 mg/kg/day<br><br>Treatment	$IL10^{-/-}$ C57BL/6j mice	**Gastrointestinal loads post *C. jejuni* infection**<br>• Fecal *C. jejuni* numbers were lower in the UroA treatment group compared to the placebo cohort (*P* <0.01-0.001) on days 2, 3 and 4 post infection.<br>• In the ileum, median loads of *C. jejuni* bacteria were almost two log orders of magnitude lower in the UroA group versus the placebo group (*P* <0.05). Not significant in duodenum, stomach, and colon.<br>• Ileal pathogen burdens were lower following UroA versus placebo application to *C. jejuni* infected mice.<br>**Clinical course of the infection**<br>• UroA cohort displayed lower clinical scores as compared to placebo counterparts as early as day 2 post infection (*P* <0.01-0.001).<br>• On day 6, UroA treated animal exhibited less pronounced clinical signs (*P* <0.01-	[30]

0.001) compared to the more severe *C. jejuni* disease of the placebo group.

- 37.5% of mice from UroA cohort were clinically uncompromised and were lacking any signs of disease.

Large intestinal lengths

- Mice from both placebo and UroA groups displayed shorter colons compared to naïve animals (*P* <0.001) but with longer lengths measured in the UroA compared to the placebo group.

Histopathology scoring of hematoxylin and eosin-stained colonic paraffin sections

- UroA treated mice has less pronounced histopathological changes versus placebo treated mice on day 6 post infection (*P* <0.05).

Immunohistochemical staining

- Lower numbers of apoptotic epithelial cell counts compared to placebo treated mice on day 6 post infection (*P* <0.001).

In situ immunohistochemistry

- UroA group showed lower numbers of colonic macrophages and monocytes (*P* <0.01) and of T lymphocytes (*P* <0.001) versus placebo treated mice.
- *C. jejuni* induced increases in regulatory T cells and B lymphocytes within the large intestines were comparable in both treatment cohorts (*P* <0.001).

Intestinal pro-inflammatory mediator secretion and interferon-γ (IFN-γ) concentration

				• Lower concentrations were found in both colonic ($P < 0.05$), ileal explants ($P < 0.001$), and ileal tumor necrosis factor-α (TNF-α) protein levels ($P < 0.05$) taken from UroA group versus placebo treated mice.<br>• No increase of monocyte chemoattractant protein-1 (MCP-1) ($P < 0.05$) and nitric oxide concentrations ($P < 0.001$) in the ileum of UroA treated mice.<br>**<u>Extra-intestinal pro-inflammatory mediators</u>**<br>• No increase of IFN-γ concentrations were found in the lungs of the UroA treated mice ($P < 0.01$)<br>• Elevated IFN-γ concentrations in liver and kidney samples were found from mice of both cohorts ($P < 0.01$-0.001).	
**Osteogenic activity**<br>**Anti-inflammatory**	**Sham**<br><br>OVX<br><br>OVX + UroA (10 mg/kg)<br><br>OVX + UroA (20 mg/kg)	**8 weeks**<br>10 mg/kg<br>20 mg/kg<br><br>Treatment	C57BL/6 female mice	**<u>Ovariectomy-induced bone loss</u>**<br>• Three-dimensional (3D) image reconstruction showed that UroA administration significantly reduced the extensive trabecular bone loss in the distal femur compared with that in the nontreated mice.<br>• No apparent side effects of UroA on body weight or tissue damage in the major metabolic organs (liver and kidneys) were observed.<br>• 10 mg/kg UroA treatment increased bone mineral density (BMD) (0.088±0.007 g/cm^3 vs 0.052±0.005 g/cm^3 and decreased trabecular separation (Tb.Sp) (0.368±0.015 mm vs 0.555±0.006 g/cm^3) compared with the corresponding values in the OVX	[31]

group.

Histological analysis

- Tb.N was higher in the UroA-treated groups than in the OVX group.
- The surface osteoclast number in cancellous bone of the distal femur was dramatically lower in the UroA-treated groups than in the OVX group.
- UroA suppressed osteoclast formation in vivo.
- Fluorescence intensity of NFATc1 was decreased by UroA administration.

Bone histomorphometry

- UroA treatment decreased ES/BS in vivo relative to OVX group.
- N.Ob/BS (N/mm) was significantly increased in UroA-treated group.

TNF-α, IL-1β, IL-6, and IL-10

- Secretion levels of TNF-α, IL-1β, and IL-6 were markedly downregulated in the 20 mg/kg UroA group compared with the OVX group and 10 mg/kg UroA group.
- IL-10 steadily increased at 20 mg/kg (p <0.01) and 10 mg/kg (p <0.05) UroA treatment groups compared to OVX group.

CTX-1 level

- UroA treatment reduce CTX-1 level in a concentration-dependent manner.

RANKL/OPG ratio

- UroA treatment decreased the RANKL/OPG ratio.
- 10 mg/kg UroA treated group vs OVX

				group (p <0.05).<br>• 20 mg/kg UroA treated group vs OVX group (p <0.01).	
**Anti-inflammatory**	**Control**<br><br>D-gal induced ageing model<br><br>D-gal + 150 mg/kg UroB<br><br>D-gal +100 mg/kg UroB<br><br>D-gal + 50 mg/kg UroB<br><br>**Control**<br><br>150/kg UroB<br><br>300 mg/kg UroB<br><br>450 mg/kg UroB<br><br>**2 months old mice control**<br><br>2 months old mice + 150 mg/kg UroB	**Not reported**<br>150 mg/kg/d<br>100 mg/kg/d<br>50 kg/mg/d<br><br>**4 weeks**<br>150 mg/kg/d<br>300 mg/kg/d<br>450 mg/kg/d<br><br>**2 months**<br>150 mg/kg/d<br><br>Treatment	C57BL/6 male mice	**<u>MDA level</u>**<br>• UroB significantly lower MDA level in UroB treated groups.<br>**<u>CAT, SOD, T-AOC, and GSH-Px</u>**<br>• Alterations induced by D-gal were restored in UroB treated groups.<br>**<u>IL-6, TNF-α, IFN-γ, and IL-1β</u>**<br>• Increased in UroB supplementation in ageing mice compared to D-gal treated ageing mice group.<br>**<u>IL-4 level</u>**<br>• Administration of UroB significantly restored its level.<br>**<u>AGE content in serum and small intestine</u>**<br>• UroB supplementation suppressed the increase of AGE content.<br>**<u>IgA and sIgA levels in serum and small intestine</u>**<br>• Significant decrease of IgA and sIgA levels in the serum and small intestine of UroB supplementation group (p <0.01 or p <0.05).<br>**<u>Effect of UroB on pathological changes of the small intestine</u>**<br>• Administration of UroB markedly ameliorated collagen accumulation in the small intestine.<br>• UroB treatment restores D-gal induced reduction of intestinal ZO-1, occluding, and MUC2 levels.	[32]

	**12 months old mice control**<br><br>12 months old mice + 150 mg/kg UroB			**<u>TLR4, IRAK4, TRAK6, IKKβ, NF-κbp65, and HMGB1</u>**<br>• UroB administration markedly suppressed the expression of these proteins compared with that in D-gal treated mice (*p* <0.05 or *p* <0.01).<br>**<u>Toxicity studies</u>**<br>• Overdose of UroB had no impact on barrier integrity and microbiota alterations in normal mice (up to 450 mg/kg).<br>**<u>Effect of UroB on the small intestine in natural ageing mice</u>**<br>• CAT, SOD, T-AOC, and GSH-Px in both young and naturally senile mice treated with UroB showed increased in levels.<br>• mRNA expression of TLR4, IRAK4, TRAF4, IKKβ, NF-κbp65, and HMGB1 was decreased in UroB treated 12 months old mice group compared with UroB treated 2 months old mice group.<br>• Decreased levels of inflammatory cytokines, AGE content, and levels of IgA in serum and sIgA in the small intestine, ameliorated small intestine pathology found in both UroB treated young and senile mice group.	
**Anti-inflammatory**	**Vehicle**<br><br>UroA<br><br>UAS03	**12 hourly**<br>**6 hourly pre-induction**<br>**4th and 6th day post induction**<br>20 mg/kg	C57BL/6<br><br>Nrf2$^{-/-}$<br><br>AhR$^{-/-}$	**<u>LPS-induced colitis</u>**<br>• UroA/UAS03 treatment significantly reduced the LPS-induced increase in serum IL-6 and TNF-α levels.<br>**<u>2,4,6-Trinitrobenzenesulfonic (TNBS) induced colitis</u>**	[33]

	Treatment<br>Preventive<br>Treatment	mice	• Oral treatment with UroA/UAS03 (20mg/kg at 12-hour intervals) significantly protected from TNBS induced body weight loss, reduced disease activity index (DAI) score and intestinal permeability.<br>• UroA/UAS03 treatment and pre-treatment significantly protected from TNBS-induced colon shortening and reduced weight to length ratio, as well as reduced neutrophil infiltration as evident from myeloperoxidase (MPO), IL-6, TNF-α, CXCL1, and IL-1β compared to vehicle treatment.<br>• H&E analysis of colon sections showed significantly less tissue damage and inflammation scores.<br>• Uro/UAS03 also protected from TNBS-induced downregulation of Cldn4 in the colons of these mice.<br>**<u>Dextran sodium sulfate (DSS) induced colitis</u>**<br>• UroA/UAS03 significantly protected from 3% DSS induced acute colitis. Decreased DAI scores during the disease progression, protected from shortening of colons, decreased gut permeability and reduced inflammation compared to vehicle treatment. At the end of experiment on day 15.<br>• Treatment with UroA/UAS03 significantly protected from chronic DSS-induced colitis as evident from decreased gut permeability, reduced shortening of

colons, increased colon weight/length ratio, reduced inflammation (serum IL-6, IL-1β, TNF-α as well as colonic tissue MPO levels)

AhR-Nrf2 pathways in barrier function

- Treatment of $Nrf2^{-/-}$ mice with UroA/UAS03 failed to restore body weight loss caused by TNBS-induced colitis, protect from shortening of colons, and it did not enhance barrier function. UroA/UAS03 enhanced gut barrier integrity requires the expression of Nrf2. UroA/UAS03 partially reduced serum inflammatory mediators such as IL-6 and TNF-α levels in $Nrf^{-/-}$ mice.
- Treatment with UroA/UAS03 failed to protect from shortening of colon lengths, correct the barrier dysfunction, failed to reduce IL-6 and slight reduced the TNF-α in in $AhR^{-/-}$ compared to wild type mice.

AhR-Nrf2 pathways in macrophages

- UroA/UAS03 treatment significantly upregulated Nrf2 expression and induced its nuclear translocation, as well as upregulation of Nrf2-target genes such as HO1 expression in macrophages.
- UroA/UAS03 mediated down regulation of LPS-induced IL-6 production in macrophages from wild type, $Nrf2^{-/-}$ (TNBS model) and $AhR^{-/-}$ mice.
- UroA/UAS03 reduced the NF-κB in an AhR-dependent manner in macrophages.

28-day repeated dose toxicity study

				• UroA or UAS03 did not exhibit any signs of toxicity as evident from no observed changes in their body weights, CBC counts as well as serum ALT and AST levels.	
**Musculoskeletal protective**	**Control**<br><br>UroA	**<u>Prevention study</u>**<br>**Rats – 34 weeks**<br><br>**<u>Intervention study</u>**<br>**Rats – 6 weeks**<br><br>**<u>Quantification of mitophagy in muscle and assessment of exercise capacity</u>**<br>**Rats – 24 weeks**<br><br><u>Prevention study</u><br>Rats – 50 mg/kg/d<br><br><u>Intervention study</u><br>Rats – 50 mg/kg/d<br><br><u>Quantification of mitophagy in muscle and assessment of exercise capacity</u><br>Rats – 25 mg/kg/d	Sprague-Dawley male rats	**<u>Prevention of age-related muscle decline</u>**<br>• Chronic UroA treatment did not affect body weight gain or the evolution of fat and lean body mass. UroA treatment resulted in a robustly increased muscle function measured at 22 and 24 months of age as compared to that in the control HFD-fed mice, in spite of the absence of changes in muscle mass, as manifested by a 9% greater grip strength and a 57% greater level of spontaneous exercise measured using the running wheel.<br>**<u>Intervention of age-related muscle decline</u>**<br>• UroA group has an average 42% greater running endurance compared to basal measurement before treatment. These mice fed with either control or UroA containing NCD, neither body weight nor body composition was affected.<br><br>**<u>Quantification of mitophagy in muscle and assessment of exercise capacity</u>**<br>**<u>Muscle function</u>**<br>• UroA treatment resulted in a running capacity 65% greater than the control group.<br>**<u>Quantification of mitophagy in muscle</u>**	[34]

				• UroA stimulated autophagy in gastrocnemius muscle. Notably, a greater ratio of LC3-II to LC3-II and the concomitant lower p62/SQSTM levels, as well as a general tendency for a higher expression of autophagy (*Becn1*, *Ulk1*, *Pik3c3*, *Atg8l*, *p62*, *Atg5*, *Atg7*, *Atg12*, *Lc3b* and *LAMP2*) and mitophagy (*Park2*) transcripts.<br>• UroA also induced the phosphorylation of AMPK in muscle tissue.<br>• No substantial differences in transcript levels of the myosin heavy chain types.<br>• UroA did not change mtDNA/nuDNA ratio or respiratory complexes abundance at the protein level in the gastrocnemius.<br>• The evaluation of OXHPHOS complexes and activity showed a lower amount of assembled CI and tendency for greater CII activity in the UroA treated versus control group.<br>• UroA did not affect ROS levels in the muscle as revealed by 4-hydroxynonenal (4-HNE) quantification.<br>• A greater level of ubiquitinated mitochondrial proteins in the gastrocnemius in the UroA treated group compared to control group.	
**Musculoskeletal protective**	**Placebo**<br><br>UroA	**16 weeks**<br>10 mg/kg body weight	C57BL/6 male mice	**<u>Onset of muscular ageing in response</u>**<br>• UroA supplementation was safely tolerated.<br>• At 28 weeks old, murine skeletal muscle	[35]

	Nicotinamide Riboside			tissues (vastus lateralis and gastrocnemius) supplemented with placebo showed significantly elevated ageing markers P16, 8-hydroxy-2'-deoxyguanosine (8-OHdG) and Ataxia-Telangiectasia Mutated (ATM) compared to skeletal muscle tissues of young C57BL/6 mice (8 weeks old). In mice supplemented with UroA, induction of age-related markers were significantly blunted.<br>**<u>ATP and NAD^+ levels in murine skeletal muscle</u>**<br>• In vivo ^{31P}NMR spectroscopy revealed elevated total ATP in the skeletal muscle in response to UroA supplementation. Specifically, α-ATP and γ-ATP levels were increased.<br>• UroA supplementation increased skeletal muscle NAD^+ levels and NAD^+/NADH ratio significantly in 28 weeks-old mice. The increased levels of NAD^+ was comparable to the effect obtained by supplementing nicotinamide riboside, at a five-fold higher dose. UroA did not influence NADH level in the muscle of supplemented mice.	
**Musculoskeletal protective**	Control<br><br>IDD<br><br>IDD+UroA	**4 weeks**<br>25 mg/kg/day<br><br>Treatment	Sprague-Dawley rats	**<u>MRI and Pfirrmann grade scores</u>**<br>• IDD+UroA treatment group had a higher T2-weighted signal intensity and a lower Pfirrmann grade than the IDD group.<br>• IDD+UroA+CompC treatment group had a lower T2-weighted signal intensity and a	[36]

	IDD+UroA+CompC			higher Pfirrmann grade than the IDD+UroA group.<br>**<u>H&E staining and safranin O fast green staining</u>**<br>• UroA treatment significantly reduced disruption of the disc structure and fibrosis of NP tissue.<br>• UroA treatment group partially retained the proteoglycan matrix as compared with the IDD group. The combined treatment with CompC and UroA significantly offset the protective effect of UroA alone on the intervertebral disc structure and matrix.<br>**<u>TUNEL assay</u>**<br>• UroA treatment group showed significant reduction in apoptosis of the intervertebral disc cells as compared with the IDD group.<br>• The CompC and UroA co-treatment group greatly offset the effect of UroA on the apoptosis of intervertebral disc cells.	
**Musculoskeletal protective**	Sham-operated (control)<br><br>Punctured and DMSO-treated mice<br><br>Punctured and UroA-treated mice	**4 weeks**<br>25 mg/kg/day<br><br>Treatment	Sprague-Dawley male rats	**<u>X-ray images</u>**<br>• UroA group showed no significant disc space narrowing.<br>**<u>Disc height index (DHI)</u>**<br>• A sligh decline was noted in the UroA group from 0.116±0.009 to 0.086±0.025, compared to IDD group which declined from 0.123±0.021 to 0.065±0.016.<br>**<u>MRI and Pfirrmann grade scores</u>**<br>• T2-weighted signal intensity was markedly higher and the distinction between the nucleus and annulus was	[37]

				clearer in the UroA group than in IDD group.<br>• The Pfirrmann scores were lower in the UroA treatment group than in the IDD group.<br>**H&E staining**<br>• UroA treatment markedly alleviated disc destruction compared with that in the IDD group.<br>**Alcian blue staining**<br>• UroA treatment group showed deep blue in the NP (inner nucleus pulposus) and inner layers of the AF (annulus fibrosis) in the UroA group, indicating pronounced expression of proteoglycan and collagen, compared to that in the IDD group.	
**Musculoskeletal protective**	Control<br><br>IDD<br><br>IDD +UroA	**4 weeks**<br>25 mg/kg<br><br>Treatment	Sprague-Dawley rats	**Disc height index (DHI)**<br>• DHI of the UroA group (0.068 ± 0.003) was significantly higher than that of the IDD group (p <0.01)<br>**Pfirrmann grade (indicate the IVD degeneration)**<br>• Pfirrmann grade scores of the UroA group were lower than those of the IDD group at 4 weeks (p <0.01).<br>**Histological analysis**<br>• UroA group (still have small number of nucleus-pulposus (NP) cells and ECM); increase histological score (p <0.01).<br>• IDD group (well-structured IVD tissue destroyed, and NP tissue almost disappeared).<br>• UroA protected proteoglycan matrix from	[38]

				decreasing and had a higher level compared to IDD group; more NP chondrocytes in UroA than IDD group.<br>**<u>Collagen type II and aggrecan</u>**<br>• Higher than that of the IDD group after treatment with UroA for 4 weeks indicates that UroA treatment can delay the downward trend of collagen type II and aggrecan (*p* <0.05).	
**Musculoskeletal protective**	**Control group (wild-type C57BL/10 mice**)<br><br>mdx group<br><br>mdx mice with UroA<br><br>mdx mice with UroA and Colchicine<br><br>**Wild type BL10 mice (control group)**<br><br>mdx mice group treated with<br><br>DMSO<br><br>UroA	**10 weeks**<br>50 mpk/day<br><br>Treatment	C57BL/10ScSn or C57BL/10ScSn-Dmd^{mdx}J male mice<br><br>mdx/Utr$^{-/-}$ male and female mice<br><br>cardiotoxin (CTX)-damaged mdx male and female mice	**<u>Expression of mitophagy-related genes</u>**<br>• UroA increased the expression of mitophagy-related genes (*Pink1*; *Park2*; *Bnip3*) and, to a lesser extent, of autophagy transcripts (*Sqstm1*; *Becn1*) in the proximal forelimb muscle of mdx mice.<br>**<u>Western blot of mitochondrial phosphor-S65-ubiquitin, BNIP3, PARKIN, and VDAC</u>**<br>• mdx mice showed reduced protein among of mitochrondrial phospho-S65-ubiquitin, Parkin, and BNIP3.<br>• The quantity of these mitophagy markers was increased with UroA treatment.<br><br>**<u>Western blot of mitochondrial LC3-II and VDAC</u>**<br>• UroA increased the quantity of mitochondrial LC3-II.<br>• The co-treatment of UroA with colchicine further induce its accumulation in the mitochondria.<br>**<u>Western blot of LC3I/II and HSP90 of proximal forelimb muscles</u>**	[39]

- HSP90 remained unchanged among groups.
- Increased LC3-II/LC3-I ratio in mdx mice.
- UroA was not able to further increase the expression of LC3-II/LC3-I

mtDNA/nDNA ratio

- UroA increased the mtDNA/nDNA ratio comparable to control group.

ATP (adenosine 5′-triphosphate) quantity of proximal forelimb muscles

- mdx group showed diminished ATP quantity.
- UroA treatment increased ATP amount.

Citrate synthase activity

- UroA treatment led to a robust elevation of citrate synthase activity.

Transcript expression of mitophagy (*Pink1*, *Park2*, *Park7*, and *Bnip3*) and autophagy (*Sqstm1* and *Becn1*) genes in MuSc (muscle stem cell) isolated from hindlimbs

- UroA treatment of mdx mice re-established the expression of these mitophagy genes to physiological extent in MuScs.

mRNA expression of stemness (*Pax7* and *Myod*), predifferentiation (*Myog*), and senescence (*p21*) markers in MuSCs from mice treated

- UroA increased the expression of the stemness marker *Pax7* and the early myogenic marker *Myod* while decreased the expression of the differentiation marker *Myog* and of the cyclin-dependent

kinase inhibitor and senescence activator *p21* in MuSCs of dystrophic animals.

Pax7, laminin and DAPI staining in TA muscles

- UroA-treated mdx mice had more MuSCs compared with untreated mdx mice.

FACS-based quantification of freshly isolated MuSCs from hindlimb muscles

- UroA fed mice group have normalized the muscle weight compared to mdx mice.

MuSC transplantation assay

- Mice receiving UroA-treated MuSCs had reduced muscle inflammation, increase average cross-sectional area of muscle fibers, and greater expression of embryonic myosin heavy chain (eMyHC).

CD45 staining of infiltrated inflammatory cells in TA (tibialis anterior) muscles

- UroA treatment reduced the number of inflammatory cells in muscle, decreased signal of the general immune cell marker CD45.

Western blot of structural proteins, α-dystrobrevin (α-DB) and β-dystroglycan (β-DG) from proximal forelimb muscles

- UroA increased the expression of the structural proteins α-dystrobrevin and β-dystroglycan.

Evans blue staining of quadriceps and gastrocnemius muscles

- UroA treatment reduced permeability of Evans blue in the gastrocnemius and quadriceps muscles.

Pre- and post-exercise creatine kinase amount

- UroA reduced the amount of creatine kinase in both untrained and posttrained mdx animals.

Percentage of muscle fibers with centralized nuclei in TA muscles

- UroA-treated mdx mice has lower percentage of fibers with centralized nuclei in the tibialis anterior muscles.

Average CSA of TA muscle fibers and distribution of muscle fiber minimal Feret diameter of mice

- UroA-treated mdx mice showed increased in muscle fiber CSA, and the enhanced muscle fiber size continued to show uniformity as manifested by the distribution of minimal Feret diameter.

Fibrosis and degeneration of the diaphragm

- Fibrosis and degeneration of the diaphragm was diminished after UroA supplementation in mdx mice.

Masson's trichrome staining

- UroA treatment reduced cardiac muscle fibrosis and hypertrophy compared with untreated dystrophic animals.

Grip strength normalized to bodyweight and uphill running distance

- UroA treatment improved the muscle strength of mdx mice.
- UroA treatment increased running performance.

Isometric force expressed as force per CSA

				**versus stimulation time relationship in extensor digitorum longus (EDL) muscles and Maximal isometric force in EDL developed during a 300-ms tetanus stimulated maximally (125Hz)**<br>• UroA treatment showed increase in the tetanic force and resistance to fatigue measured ex vivo in isolated ex<br>**Kaplan-Meier survival curves of mdx/Utr$^{-/-}$**<br>UroA treatment improved the survival rate of DKO dystrophic animals.	
**Osteogenic activity**	**Sham**<br><br>Bone defect<br><br>Bone defect + UroA	**Unknown**<br>Unknown<br><br>Treatment	BALB/c mice	**Serum osteocalcin level**<br>• The serum osteocalcin level in UroA treated group (7.84±0.19 pg/mL) was higher than that of bone defect mice group (2.93±0.13 pg/mL) (p <0.01).<br>**CTX level**<br>• UroA treated group (61.7±3.1 ng/mL) significantly reduced the serum CTX level compared to bone defect mice group (152.9±5.2 ng/mL) (p <0.01).<br>**Body weight and organ indices**<br>• No difference in the body weight, heart, renal, and lung induces among sham, bone defect, and UroA treated group.<br>• Spleen index of UroA treated group has increased value.<br>**mRNA levels of osteogenic factors**<br>• UroA significantly increased the mRNA levels of Sox9, Col2a1, Runx2, MMP13, and osterix (p <0.01).<br>**Histopathological changes**<br>• UroA was shown to promote new bone	[40]

				formation and angiogenesis.<br>• UroA increased the histopathologic score.<br>**<u>Morphological changes and BMD in femur tissue</u>**<br>• UroA treatment (p <0.01) significantly increased the bone defect score compared to sham and bone defect mice.<br>• UroA enhanced the femoral BMD in bone defect mice.<br>**<u>Wnt3a and GSK3β protein levels</u>**<br>• Wnt3a and GSK3β protein levels were significantly increased in the UroA treated and bone defect groups.	
**Radioprotective activity**	**Control**<br><br>IR<br><br>IR + 0.4 mg/kg UroA<br><br>IR + 2 mg/kg UroA<br><br>IR + 10 mg/kg UroA<br><br>**Control**<br><br>IR<br><br>IR + 2 mg/kg UroA	**48 hours, 24 hours, 1 hour prior to and 24 hours after 9.0Gy total body irradiation (TBI)**<br>0.4 mg/kg<br>2 mg/kg<br>10 mg/kg<br><br>**48 hours, 24 hours, 1 hour prior to and 24 hours after 9.0Gy total body irradiation (TBI)**<br>2 mg/kg	C57BL/6 male mice	**<u>Survival rate of irradiated mice</u>**<br>• All three doses of UroA improve the survival rate of irradiated mice at 5 days after 9.0Gy TBI<br>• 2 mg/kg UroA treated group performed the best (p <0.001).<br>• 10% of the IR mice survived in the 0.4 mg/kg UroA treated group. (Average survival days are 4.8 days)<br>• 70% of the IR mice survived in the 2 mg/kg UroA treated group. (Average survival days are 6 days)<br>• 40% of the IR mice survived in the 10 mg/kg UroA treated group. (Average survival days are 5.4 days)<br>**<u>Histological analysis</u>**<br>• UroA treated group showed more survival crypts in the small intestine (p <0.001).<br>• UroA treatment rescue the loss of villi	[41]

		height reduced by 9.0Gy TBI.<br>• The loss of $Lgr5^+$ positive cells was relieved by UroA treatment compared to that of IR group.<br>**Axin2 protein expression**<br>• UroA treatment alleviate the loss of Axin2.<br>**Ki67 expression**<br>• UroA treatment significantly promote the crypts regeneration with more Ki67 expression.<br>**Lysozyme**<br>• UroA treatment rescued the reduction of lysozyme induced by radiation.<br>**8-OHdG**<br>• UroA significantly alleviate the increase in 8-OHdG in small intestine of IR mice.<br>**P53 protein expression**<br>• The p53 in intestinal tissue was remarkably restored to the normal level by the administration of UroA.<br>**Caspase8 and caspase3**<br>• UroA prevented the upregulation of caspase8 and caspase3 observed in the small intestine of mice exposed with 9.0Gy TBI.

References

1. Gong, Z.; Huang, J.Y.; Xu, B.; Ou, Z.R.; Zhang, L.; Lin, X.H.; Ye, X.J.; Kong, X.J.; Long, D.H.; Sun, X.D., et al. Urolithin A attenuates memory impairment and neuroinflammation in APP/PS1 mice. Journal of Neuroinflammation 2019, 16, 13, doi:10.1186/s12974-019-1450-3.
2. Fang, E.F.; Hou, Y.J.; Palikaras, K.; Adriaanse, B.A.; Kerr, J.S.; Yang, B.M.; Lautrup, S.; Hasan-Olive, M.M.; Caponio, D.; Dan, X.L., et al. Mitophagy inhibits amyloid-beta and tau pathology and reverses cognitive deficits in models of Alzheimer's disease. Nature Neuroscience 2019, 22, 401-+, doi:10.1038/s41593-018-0332-9.
3. Chen, P.; Chen, F.C.; Lei, J.X.; Li, Q.L.; Zhou, B.H. Activation of the miR-34a-Mediated SIRT1/mTOR Signaling Pathway by Urolithin A Attenuates d-Galactose-Induced Brain Aging in Mice. Neurotherapeutics 2019, 16, 1269-1282, doi:10.1007/s13311-019-00753-0.
4. Chen, P.; Chen, F.; Lei, J.; Wang, G.; Zhou, B. The Gut Microbiota Metabolite Urolithin B Improves Cognitive Deficits by Inhibiting Cyt C-Mediated Apoptosis and Promoting the Survival of Neurons Through the PI3K Pathway in Aging Mice. Frontiers in Pharmacology 2021, 12, doi:10.3389/fphar.2021.768097.
5. Shen, P.X.; Li, X.; Deng, S.Y.; Zhao, L.; Zhang, Y.Y.; Deng, X.; Han, B.; Yu, J.; Li, Y.; Wang, Z.Z., et al. Urolithin A ameliorates experimental autoimmune encephalomyelitis by targeting aryl hydrocarbon receptor. EBioMedicine 2021, 64 (no pagination), doi:http://dx.doi.org/10.1016/j.ebiom.2021.103227.
6. Tan, S.; Tong, W.H.; Vyas, A. Urolithin-A attenuates neurotoxoplasmosis and alters innate response towards predator odor. Brain, Behavior, and Immunity - Health 2020, 8 (no pagination), doi:http://dx.doi.org/10.1016/j.bbih.2020.100128.
7. Chen, P.; Lei, J.X.; Chen, F.C.; Zhou, B.H. Ameliorative effect of urolithin A on d-gal-induced liver and kidney damage in aging mice via its antioxidative, anti-inflammatory and antiapoptotic properties. RSC Adv. 2020, 10, 8027-8038, doi:10.1039/d0ra00774a.
8. Wang, Y.; Huang, H.P.; Jin, Y.W.; Shen, K.Z.; Chen, X.Y.; Xu, Z.J.; Jin, B.Y.; Pan, H. Role of TFEB in autophagic modulation of ischemia reperfusion injury in mice kidney and protection by urolithin A. Food and Chemical Toxicology 2019, 131, 8, doi:10.1016/j.fct.2019.110591.
9. Zhang, Y.; Liu, M.; Zhang, Y.; Tian, M.; Chen, P.; Lan, Y.; Zhou, B. Urolithin A alleviates acute kidney injury induced by renal ischemia reperfusion through the p62-Keap1-Nrf2 signaling pathway. Phytother Res 2022, 36, 984-995, doi:10.1002/ptr.7370.
10. Guada, M.; Ganugula, R.; Vadhanam, M.; Kumar, M. Urolithin A Mitigates Cisplatin-Induced Nephrotoxicity by Inhibiting Renal Inflammation and Apoptosis in an Experimental Rat Models. Journal of Pharmacology and Experimental Therapeutics 2017, 363, 58-65, doi:10.1124/jpet.117.242420.
11. Jing, T.L.; Liao, J.Z.; Shen, K.Z.; Chen, X.Y.; Xu, Z.J.; Tian, W.J.; Wang, Y.M.; Jin, B.Y.; Pan, H. Protective effect of urolithin a on cisplatin-induced nephrotoxicity in mice via modulation of inflammation and oxidative stress. Food and Chemical Toxicology 2019, 129, 108-114, doi:10.1016/j.fct.2019.04.031.
12. Zou, D.X.; Ganugula, R.; Arora, M.; Nabity, M.B.; Sheikh-Hamad, D.; Kumar, M. Oral delivery of nanoparticle urolithin A normalizes cellular stress and improves survival in mouse model of cisplatin-induced AKI. Am. J. Physiol.-Renal Physiol. 2019, 317, F1255-F1264, doi:10.1152/ajprenal.00346.2019.
13. Li, Q.L.; Li, K.Y.; Chen, Z.Z.; Zhou, B.H. Anti-renal fibrosis and anti-inflammation effect of urolithin B, ellagitannin-gut microbial-derived metabolites in unilateral ureteral obstruction rats. J. Funct. Food. 2020, 65, 13, doi:10.1016/j.jff.2019.103748.
14. Toney, A.M.; Fan, R.; Xian, Y.B.; Chaidez, V.; Ramer-Tait, A.E.; Chung, S. Urolithin A, a Gut Metabolite, Improves Insulin Sensitivity Through Augmentation of Mitochondrial Function and Biogenesis. Obesity 2019, 27, 612-620, doi:10.1002/oby.22404.
15. Yang, J.; Guo, Y.; Henning, S.M.; Chan, B.; Long, J.; Zhong, J.; Acin-Perez, R.; Petcherski, A.; Shirihai, O.; Heber, D., et al. Ellagic Acid and Its Microbial Metabolite Urolithin A Alleviate Diet-Induced Insulin Resistance in Mice. Molecular nutrition & food research 2020, 64, e2000091, doi:http://dx.doi.org/10.1002/mnfr.202000091.
16. Zhang, Y.; Zhang, Y.; Halemahebai, G.; Tian, L.; Dong, H.; Aisker, G. Urolithin A, a pomegranate metabolite, protects pancreatic β cells from apoptosis by activating autophagy. J Ethnopharmacol 2021, 272, 113628, doi:10.1016/j.jep.2020.113628.
17. Tuohetaerbaike, B.; Zhang, Y.; Tian, Y.L.; Zhang, N.N.; Kang, J.S.; Mao, X.M.; Zhang, Y.Z.; Li, X.J. Pancreas protective effects of Urolithin A on type 2 diabetic mice induced by high fat and streptozotocin via regulating autophagy and AKT/mTOR signaling pathway. Journal of Ethnopharmacology 2020, 250, 12, doi:10.1016/j.jep.2019.112479.
18. Abdulrahman, A.O.; Alzubaidi, M.Y.; Nadeem, M.S.; Khan, J.A.; Rather, I.A.; Khan, M.I. Effects of urolithins on obesity-associated gut dysbiosis in rats fed on a high-fat diet. International journal of food sciences and nutrition 2021, http://dx.doi.org/10.1080/09637486.2021.1886255, 1-12, doi:http://dx.doi.org/10.1080/09637486.2021.1886255.

19. Zhang, Y.; Aisker, G.; Dong, H.; Halemahebai, G.; Zhang, Y.; Tian, L. Urolithin A suppresses glucolipotoxicity-induced ER stress and TXNIP/NLRP3/IL-1β inflammation signal in pancreatic β cells by regulating AMPK and autophagy. Phytomedicine 2021, 93, 153741, doi:10.1016/j.phymed.2021.153741.
20. Zhao, W.; Wang, L.; Haller, V.; Ritsch, A. A Novel Candidate for Prevention and Treatment of Atherosclerosis: Urolithin B Decreases Lipid Plaque Deposition in apoE(-/-) Mice and Increases Early Stages of Reverse Cholesterol Transport in ox-LDL Treated Macrophages Cells. Mol Nutr Food Res 2019, 63, e1800887, doi:10.1002/mnfr.201800887.
21. Dahiya, N.R.; Chandrasekaran, B.; Kolluru, V.; Ankem, M.; Damodaran, C.; Vadhanam, M.V. A natural molecule, urolithin A, downregulates androgen receptor activation and suppresses growth of prostate cancer. Mol Carcinog 2018, 57, 1332-1341, doi:10.1002/mc.22848.
22. Lv, M.Y.; Shi, C.J.; Pan, F.F.; Shao, J.; Feng, L.; Chen, G.; Ou, C.; Zhang, J.F.; Fu, W.M. Urolithin B suppresses tumor growth in hepatocellular carcinoma through inducing the inactivation of Wnt/β-catenin signaling. J Cell Biochem 2019, 120, 17273-17282, doi:10.1002/jcb.28989.
23. Zhou, B.; Wang, J.; Zheng, G.; Qiu, Z. Methylated urolithin A, the modified ellagitannin-derived metabolite, suppresses cell viability of DU145 human prostate cancer cells via targeting miR-21. Food Chem Toxicol 2016, 97, 375-384, doi:10.1016/j.fct.2016.10.005.
24. Tang, L.; Mo, Y.; Li, Y.; Zhong, Y.; He, S.; Zhang, Y.; Tang, Y.; Fu, S.; Wang, X.; Chen, A. Urolithin A alleviates myocardial ischemia/reperfusion injury via PI3K/Akt pathway. Biochem Biophys Res Commun 2017, 486, 774-780, doi:10.1016/j.bbrc.2017.03.119.
25. Wang, Y.; Jasper, H.; Toan, S.; Muid, D.; Chang, X.; Zhou, H. Mitophagy coordinates the mitochondrial unfolded protein response to attenuate inflammation-mediated myocardial injury. Redox biology 2021, 45, 102049-102049, doi:10.1016/j.redox.2021.102049.
26. Albasher, G.; Alkahtani, S.; Al-Harbi, L.N. Urolithin A prevents streptozotocin-induced diabetic cardiomyopathy in rats by activating SIRT1. Saudi Journal of Biological Sciences 2022, 29, 1210-1220, doi:https://doi.org/10.1016/j.sjbs.2021.09.045.
27. Gao, H.; Huang, X.; Tong, Y.; Jiang, X. Urolithin B improves cardiac function and reduces susceptibility to ventricular arrhythmias in rats after myocardial infarction. Eur J Pharmacol 2020, 871, 172936, doi:10.1016/j.ejphar.2020.172936.
28. Zheng, D.; Liu, Z.; Zhou, Y.; Hou, N.; Yan, W.; Qin, Y.; Ye, Q.; Cheng, X.; Xiao, Q.; Bao, Y., et al. Urolithin B, a gut microbiota metabolite, protects against myocardial ischemia/reperfusion injury via p62/Keap1/Nrf2 signaling pathway. Pharmacol Res 2020, 153, 104655, doi:10.1016/j.phrs.2020.104655.
29. Saha, P.; Yeoh, B.S.; Singh, R.; Chandrasekar, B.; Vemula, P.K.; Haribabu, B.; Vijay-Kumar, M.; Jala, V.R. Gut Microbiota Conversion of Dietary Ellagic Acid into Bioactive Phytoceutical Urolithin A Inhibits Heme Peroxidases. PLoS One 2016, 11, e0156811, doi:10.1371/journal.pone.0156811.
30. Mousavi, S.; Weschka, D.; Bereswill, S.; Heimesaat, M.M. Preclinical evaluation of oral urolithin-a for the treatment of acute campylobacteriosis in campylobacter Jejuni infected microbiota-depleted il-10^{-/-} mice. Pathogens 2021, 10, 1-16, doi:http://dx.doi.org/10.3390/pathogens10010007.
31. Tao, H.; Li, W.; Zhang, W.; Yang, C.; Zhang, C.; Liang, X.; Yin, J.; Bai, J.; Ge, G.; Zhang, H., et al. Urolithin A suppresses RANKL-induced osteoclastogenesis and postmenopausal osteoporosis by, suppresses inflammation and downstream NF-κB activated pyroptosis pathways. Pharmacological Research 2021, 174, 105967, doi:https://doi.org/10.1016/j.phrs.2021.105967.
32. Chen, P.; Chen, F.; Lei, J.; Zhou, B. Gut microbial metabolite urolithin B attenuates intestinal immunity function in vivo in aging mice and in vitro in HT29 cells by regulating oxidative stress and inflammatory signalling. Food & Function 2021, 12, 11938-11955, doi:10.1039/D1FO02440J.
33. Singh, R.; Chandrashekharappa, S.; Bodduluri, S.R.; Baby, B.V.; Hegde, B.; Kotla, N.G.; Hiwale, A.A.; Saiyed, T.; Patel, P.; Vijay-Kumar, M., et al. Enhancement of the gut barrier integrity by a microbial metabolite through the Nrf2 pathway. Nature Communications 2019, 10, 89, doi:10.1038/s41467-018-07859-7.
34. Ryu, D.; Mouchiroud, L.; Andreux, P.A.; Katsyuba, E.; Moullan, N.; Nicolet-Dit-Félix, A.A.; Williams, E.G.; Jha, P.; Lo Sasso, G.; Huzard, D., et al. Urolithin A induces mitophagy and prolongs lifespan in C. elegans and increases muscle function in rodents. Nat Med 2016, 22, 879-888, doi:10.1038/nm.4132.
35. Ghosh, N.; Das, A.; Biswas, N.; Gnyawali, S.; Singh, K.; Gorain, M.; Polcyn, C.; Khanna, S.; Roy, S.; Sen, C.K. Urolithin A augments angiogenic pathways in skeletal muscle by bolstering NAD+ and SIRT1. Scientific Reports 2020, 10, 20184, doi:10.1038/s41598-020-76564-7.
36. Lin, J.; Zhuge, J.; Zheng, X.; Wu, Y.; Zhang, Z.; Xu, T.; Meftah, Z.; Xu, H.; Wu, Y.; Tian, N., et al. Urolithin A-induced mitophagy suppresses apoptosis and attenuates intervertebral disc degeneration via the AMPK signaling pathway. Free Radic Biol Med 2020, 150, 109-119, doi:10.1016/j.freeradbiomed.2020.02.024.
37. Liu, H.; Kang, H.; Song, C.; Lei, Z.; Li, L.; Guo, J.; Xu, Y.; Guan, H.; Fang, Z.; Li, F. Urolithin A Inhibits the Catabolic Effect of TNFα on Nucleus Pulposus Cell and Alleviates Intervertebral Disc Degeneration in vivo. Front Pharmacol 2018, 9, 1043, doi:10.3389/fphar.2018.01043.
38. Shi, P.Z.; Wang, J.W.; Wang, P.C.; Han, B.; Lu, X.H.; Ren, Y.X.; Feng, X.M.; Cheng, X.F.; Zhang, L. Urolithin a

alleviates oxidative stress-induced senescence in nucleus pulposus-derived mesenchymal stem cells through SIRT1/PGC-1α pathway. World J Stem Cells 2021, 13, 1928-1946, doi:10.4252/wjsc.v13.i12.1928.
39. Luan, P.; D'Amico, D.; Andreux, P.A.; Laurila, P.P.; Wohlwend, M.; Li, H.; Imamura de Lima, T.; Place, N.; Rinsch, C.; Zanou, N., et al. Urolithin A improves muscle function by inducing mitophagy in muscular dystrophy. Sci Transl Med 2021, 13, doi:10.1126/scitranslmed.abb0319.
40. Liu, J.; Ma, L.; Dong, W.; Du, G.; Dang, X. Effect of Urolithin A on Bone Repair in Mice with Bone Defects. Tissue Engineering and Regenerative Medicine 2022, 19, 151-159, doi:10.1007/s13770-021-00382-9.
41. Zhang, Y.; Dong, Y.; Lu, P.; Wang, X.; Li, W.; Dong, H.; Fan, S.; Li, D. Gut metabolite Urolithin A mitigates ionizing radiation-induced intestinal damage. J Cell Mol Med 2021, 25, 10306-10312, doi:10.1111/jcmm.16951.
